# Role of FGFR2c and Its PKCε Downstream Signaling in the Control of EMT and Autophagy in Pancreatic Ductal Adenocarcinoma Cells

**DOI:** 10.3390/cancers13194993

**Published:** 2021-10-05

**Authors:** Danilo Ranieri, Luisa Guttieri, Salvatore Raffa, Maria Rosaria Torrisi, Francesca Belleudi

**Affiliations:** 1 Department of Clinical and Molecular Medicine, Sapienza University of Rome, 00161 Rome, Italy; danilo.ranieri@uniroma1.it (D.R.); luisa.guttieri@uniroma1.it (L.G.); salvatore.raffa@uniroma1.it (S.R.); 2 Laboratory of Ultrastructural Pathology, Unit of Medical Genetics and Advanced Cellular Diagnostics, Department of Diagnostic Sciences, Sant’Andrea University Hospital, 00189 Rome, Italy

**Keywords:** FGFR2c, PDAC, epithelial–mesenchymal transition (EMT), autophagy, PKCε

## Abstract

**Simple Summary:**

This work aims to assess the contribution of the aberrant expression of the mesenchymal FGFR2c and the triggering of the downstream PKCε signaling in selected cell lines from pancreatic ductal adenocarcinoma (PDAC), a malignancy characterized by acquired EMT signature and deregulated autophagy. The results show that: *(i)* FGFR2c expression appears to impact on cell responsiveness to FGF2 in terms of intracellular signaling activation and upregulation of EMT expression profile and *(ii)* PKCε signaling, activated in PANC-1 cell line, expressing high levels of FGFR2c, is involved in both receptor-mediated enhancement of EMT and inhibition of autophagy. Overall, this study suggests that PKCε could be a possible therapeutic target whose inactivation could contribute in counteracting tumor aggressive phenotype.

**Abstract:**

Pancreatic ductal adenocarcinoma (PDAC) is a treatment-resistant malignancy characterized by a high malignant phenotype including acquired EMT signature and deregulated autophagy. Since we have previously described that the aberrant expression of the mesenchymal FGFR2c and the triggering of the downstream PKCε signaling are involved in epidermal carcinogenesis, the aim of this work has been to assess the contribution of these oncogenic events also in the pancreatic context. Biochemical, molecular and immunofluorescence approaches showed that FGFR2c expression impacts on PDAC cell responsiveness to FGF2 in terms of intracellular signaling activation, upregulation of EMT-related transcription factors and modulation of epithelial and mesenchymal markers compatible with the pathological EMT. Moreover, shut-off via specific protein depletion of PKCε signaling, activated by high expression of FGFR2c resulted in a reversion of EMT profile, as well as in a recovery of the autophagic process. The detailed biochemical analysis of the intracellular signaling indicated that PKCε, bypassing AKT and directly converging on ERK1/2, could be a signaling molecule downstream FGFR2c whose inhibition could be considered as possible effective therapeutic approach in counteracting aggressive phenotype in cancer.

## 1. Introduction

The pancreatic ductal adenocarcinoma (PDAC) is one of the most lethal malignancies characterized by high frequency of activating mutations in *KRAS* gene [1,2]. In this context, PI3K-AKT-MTOR and Raf-MEK-ERK signaling have been described as the main RAS downstream pathways, strongly intersecting with each other, involved in the control of several oncogenic outcomes, including cell growth dysregulation, epithelial to mesenchymal transition (EMT) induction and autophagic enhancement [2,3,4,5]. Since KRAS is considered an “undruggable” signaling molecule, more and more relevance has been given to the identification of new signaling molecules, possibly bypassing RAS, whose inactivation could significantly impact on the PDAC aggressive phenotype.

PKC-mediated signaling has been described as one of the main RAS-independent pathways activated by several receptor tyrosine kinases (RTKs), including fibroblast growth factor receptors (FGFRs) [6], whose dysregulation significantly contributes to cancer development [7]. Concerning this topic, we have recently demonstrated a central contribution for the PKCε isoform in the oncogenic outcomes established by the signaling of the mesenchymal isoform of FGFR2 (FGFR2c) when expressed in the epithelial context [8,9]. Even if the aberrant expressions of FGFR2c or FGFR2 altered splicing have been previously proposed as events contributing to pancreatic carcinogenesis [10,11,12], their relevance in the establishment of cell invasion, even if extensively investigated [10,11,12], remains controversial and still to be clarified.

Further investigations are also required to establish if, in PDAC, the aberrant expression of FGFR2c can impact on autophagy, a lysosomal-associated degradative pathway whose complex crosstalk with EMT has been widely described in cancer [13]. Even if most evidence points to autophagy as survival strategy contributing to the malignant progression of PDAC [2,14,15], some findings have suggested for this process a tumor suppressive role, preventing cancer development at its early stages [15,16]. However, despite the central and context-dependent role widely proposed for autophagy in pancreatic tumors, the signaling network controlling the process has been only partially clarified [2,15,17]. The idea of a possible involvement of PKCε emerges from our recent findings, showing that this substrate contributes to the regulation of the negative crosstalk between EMT and autophagy orchestrated by FGFR2c during early steps of epidermal carcinogenesis [8]. Our hypothesis is also supported by a recent study, reporting that PKCε signaling can negatively impact on autophagy directly converging on MTOR in breast cancer cells [18].

Therefore, in light of these suggestions, in this work we aimed to further characterize the involvement of FGF/FGFR2c axis and to establish the possible role played by the downstream PKCε signaling in the control of EMT and autophagy in the context of pancreatic tumor.

## 2. Materials and Methods

### 2.1. Cells and Treatments

The human keratinocyte cell line HaCaT and the pancreatic adenocarcinoma cell line PANC-1 and MIAPaCa-2 were purchased from American Type Culture Collection (ATCC) and were cultured in Dulbecco’s modified Eagle’s medium (DMEM), supplemented with 10% fetal bovine serum (FBS) plus antibiotics. For FGFR2 and PKCε silencing, cells were stably transduced with Bek/FGFR2 shRNA (h) Lentiviral Particles (Santa Cruz Biotechnology, Inc., Santa Cruz, CA, USA; SC-29218-V) or PKCε shRNA (h) Lentiviral Particles vector (Santa Cruz; SC-36251-V) and Control shRNA Lentiviral Particles-A (Santa Cruz; SC-108080) as a control. For RNA interference and consequent specific FGFR2b or FGFR2c silencing, cells were transfected with a FGFR2b siRNA sequence (5’-AATTATATAGGGCAGGCCAAC-3’) (Qiagen, Valencia, CA, USA) or FGFR2c siRNA sequence (5’-GGAATGTAACTTTTGAGGA-3’) (Qiagen) or with a control sequence (5’-AATTCTCCGAACGTGTCACGT-3’) (Qiagen) using Lipofectamine 2000 Transfection Reagent (Invitrogen, Carlsbad, CA, USA 11668030) according to the manufacturer’s protocol. For growth factor stimulation, cells were left untreated or incubated with FGF2 (PeproTech, London, UK; BFGF 100-188) 100 ng/mL for 24 h at 37 °C. For inhibition of FGFR2 tyrosine kinase activity, cells were pre-incubated with a specific FGFR2 tyrosine kinase inhibitor, SU5402 25 µmol/L (Calbiochem, Nottingham, UK; 572 630) for 1 h before treatments with FGF2. 

### 2.2. Immunofluorescence

Cells were grown on coverslips, fixed with 4% paraformaldehyde in PBS for 30 min at 25 °C followed by treatment with 0.1 M glycine for 20 min at 25 °C and with 0.1% Triton X-100 for an additional 5 min at 25 °C to allow permeabilization. Cells were then incubated with the following primary antibodies: polyclonal antibodies anti-vimentin (1:50 in PBS; Dako, Glostrup, Denmark; M0725); mouse monoclonal anti-LC3 (1:100 in PBS, 5F10 Nanotools, Teningen, Germany; 0231) for 1 h at 25 °C and then with a goat anti-mouse IgG-Alexa Fluor 488 (1:200 in PBS, Life Technologies, Carlsbad, CA, USA; A11001) for 30 min at 25 °C. Nuclei were stained with DAPI (Sigma-Aldrich, Saint Louis, MO, USA; D9542). All fluorescence signals were analyzed by scanning cells in a series of sequential sections with an ApoTome System (Zeiss, Oberkochen, Germany) connected with an Axiovert 200 inverted microscope (Zeiss); image analysis was performed by the Axiovision software (Zeiss), and images were obtained by 3D reconstruction of the total number of the serial optical sections. Quantitative analysis of LC3-positive dots per cell was performed analyzing 100 cells for each sample in 5 different microscopy fields from 3 different experiments. Results are shown as means ± standard deviation (SD). 

### 2.3. Western Blot Analysis 

HaCaT, PANC-1 and MiaPaCa-2 cells were lysed in a buffer containing 50 mM HEPES, pH 7.5, 150 mM NaCl, 1% glycerol, 1% Triton X-100, 1.5 mM MgCl2, 5 mM EGTA, supplemented with protease inhibitors (10 g/mL aprotinin, 1 mM phenylmethylsulfonyl fluoride [PMSF], 10 µg/mL leupeptin) and phosphatase inhibitors (1 mM sodium orthovanadate, 20 mM sodium pyrophosphate, 0.5 M NaF). A range of 20 to 50 µg of total protein was resolved under reducing conditions by 8 or 12% SDS-PAGE and transferred to reinforced nitrocellulose (BA-S 83; Schleicher & Schuell, Keene, NH, USA; BA-S83). The membranes were blocked with 5% nonfat dry milk (Bio-Rad Laboratories, Hercules, CA, USA, 170-6404) in PBS 0.1% Tween 20 (Bio-Rad, 170-6531) and incubated with anti-E- cadherin (Dako, Carpinteria, CA, USA; NCH-38), anti-p-MTOR (Ser 2448; Cell Signaling, 5536S) monoclonal antibodies or anti-vimentin (M0725, Dako, Glostrup, Denmark), anti-SQSTM1 (BD Bioscience, San Josè, CA, USA, 610833), anti-LC3 (MBL, Woburn, MA, USA; PD014), anti-Bek (Santa Cruz Biotechnology; C17, sc-122), anti-p-p44/42 mitogen-activated protein kinase (MAPK) (p-ERK1/2) (Thr202/Tyr204; Cell Signaling, 9101S), anti-p-AKT (Ser 473; Cell Signaling, 9271), anti-p-S6K (ser 371, Cell Signaling, #9208), and anti p-PKCε (Ser729, Abcam, Cambridge, UK; ab63387) polyclonal antibodies followed by enhanced chemiluminescence (ECL) detection (Thermo Scientific, Rockford, IL, USA; 34580). The membranes were rehydrated by washing in PBS/Tween-20, stripped with 100 mM mercaptoethanol and 2% SDS for 30 min at 55 °C and probed again with anti-AKT (Santa Cruz Biotechnology; sc-8312), anti-p44/42 MAPK (ERK1/2) (Cell Signaling; 4695S), anti-S6K (Cell Signaling; #9202), anti-PKCε (Abcam; ab124806), anti- α/β- Tubulin (Cell Signaling; 2148S) polyclonal antibodies or anti-MTOR (Cell Signaling; 2983S), anti-ACTB (Sigma-Aldrich; A5441) monoclonal antibodies to estimate the protein equal loading. Densitometric analysis was performed using Quantity One Program version 4.6.8 (Bio-Rad). The resulting values from three different experiments were normalized, expressed as fold increase respect to the control value and reported in graph as mean values ± SD.

### 2.4. Transmission Electron Microscopy

PANC-1 and PANC-1 PKCε shRNA cells left untreated or stimulated with FGF2 for 24 h as above were washed three times in PBS and fixed with 2% glutaraldehyde (Electron Microscopy Science, 16300) in PBS for 2 h at 4 °C. Samples were postfixed with 1% osmium tetroxide in veronal acetate buffer (pH 7.4) for 1 hr at 25 °C, stained with uranyl acetate (5 mg/mL) for 1 h at 25 °C, dehydrated in acetone and embedded in Epon 812 (EMbed 812, Electron Microscopy Science). Ultrathin sections were examined unstained or poststained with uranyl acetate and lead hydroxide, under a Morgagni 268D transmission electron microscope (FEI, Hillsboro, OR, USA) equipped with a MegaView II charge-coupled device camera (SIS, Soft Imaging System GmbH, Munster, Germany) and analyzed with AnalySIS software (SIS).

### 2.5. Primers 

Oligonucleotide primers necessary for target genes and the housekeeping gene were chosen by using the online tool Primer-BLAST [19] and purchased from Invitrogen. The following primers were used: for the E-cadherin target gene: 5′-TGGAGGAATTCTTGCTTTGC-3′ (sense), 5′-CGCTCTCCTCCGAAGAAAC-3′ (antisense); for the vimentin target gene: 5′-AAATGGCTCGTCACCTTCGT-3′ (sense), 5′- AGAAATCCTGCTCTCCTCGC-3′ (antisense); for the Snail1 target gene: 5′-GCTGCAGGACTCTAATCCAGA-3′ (sense), 5′-ATCTCCGGAGGTGGGATG-3′ (antisense); for the STAT3 target gene: 5′-CAGAGATGTGGGAATGGGGG-3′ (sense), 5′-TGGCAAGGAG TGGGTCTCTA-3′ (antisense); for the FRA1 target gene: 5′-GCAGGCGGAGACTGACAAA-3′ (sense), 5′- GATGGGTCGGTGGGCTTC-3′; for FGFR2b target gene: 5′-CGTGGAAAAGAACGGCAGTAAATA-3′ (sense), 5′-GAACTATTTATCCCCGAGTGCTTG-3′ (antisense); for FGFR2c target gene: 5′-TGAGGACGCTGGGGAATATACG-3′ (sense), 5′-TAGTCTGGGGAAGCTGTAATCTCCT-3′ (antisense); for the 18S rRNA housekeeping gene: 5′-CGAGCCGCCTGGATACC-3′ (sense) and 5′-CATGGCCTCAGTTCCGAAAA-3′ (antisense). For each primer pair, we performed no-template control and no-reverse-transcriptase control (reverse transcription [RT]-negative) assays, which produced negligible signals.

### 2.6. RNA Extraction and cDNA Synthesis 

Total RNA from HaCaT, PANC-1 and MiaPaCa-2 cells was extracted using the TRIzol method (Invitrogen, 15596018) according to the manufacturer’s instructions and eluted with 0.1% diethylpyrocarbonate (DEPC)-treated water. Each sample was treated with DNase I (Invitrogen, 18068-015). The total RNA concentration was quantitated by spectrophotometry; 1 µg of total RNA was used for reverse transcription using the iScriptTM cDNA synthesis kit (Bio-Rad, 170-8891) according to the manufacturer’s instructions.

### 2.7. PCR Amplification and Real-Time Quantitation 

Real-time RT-PCR was performed using the iCycler real-time detection system (iQ5 Bio-Rad) with optimized PCR conditions. The reactions were carried out in a 96-well plate using iQ SYBR green supermix (Bio-Rad, 1708882), adding forward and reverse primers for each gene and 1 µL of diluted template cDNA to a final reaction mixture volume of 15 µL. All assays included a negative control and were replicated three times. The thermal cycling program was performed as described previously [20]. Real-time quantitation was performed with the help of the iCycler IQ optical system software, version 3.0a (Bio-Rad), according to the manufacturer’s manual. Results are reported as mean values ± SD from three different experiments in triplicate. 

### 2.8. Statistics

The data were analyzed using analysis of variance (ANOVA) to test for differences amongst all means. A Tukey’s multiple comparison test was used to determine differences between selected groups. The significance levels were defined as *p* values ≤ 0.05.

## 3. Results

### 3.1. FGFR2c Aberrant Expression Affects the Intracellular Signaling

**Figure 1:**
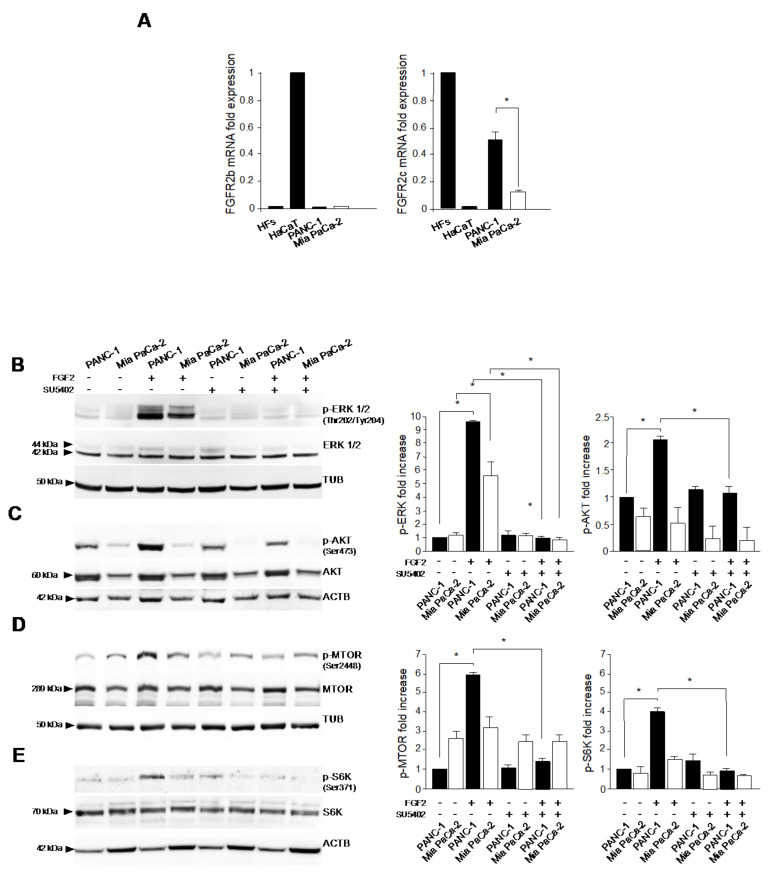
FGFR2c expression affects the susceptibility of ERK1/2 and AKT signaling to FGF2. PANC-1 and Mia PaCa−2 pancreatic tumor cell lines were left untreated or stimulated with FGF2 in the presence or absence of the FGFR2 tyrosine kinase inhibitor SU5402, as described in material and methods. (**A**) Real−time RT−PCR was performed normalizing mRNA levels respect to 18SrRNA. FGFR2c mRNA levels are significantly higher in PANC−1 cells compared to Mia PaCa−2. No appreciable levels of FGFR2b mRNA are detected in both PDAC cell lines. Human HaCaT keratinocyte cell line and normal human fibroblasts (HFs) are used as positive controls for FGFR2b and FGFR2c expression, respectively. Results are expressed as mean value ± SD (*n* = 3). ANOVA with Tukey’s multiple comparison test: * *p* ≤ 0.05. (**B**–**E**) Western blot analysis shows that the enhancement of ERK1/2 phosphorylation after FGF2 stimulation is higher in PANC−1 than in Mia PaCa−2 cells (**B**), while that of AKT was exclusively visible in PANC−1 cells (**C**). The treatment with SU5402 abrogates these effects (**B**,**C**). An increase of both MTOR and S6K phosphorylation upon FGF2 treatment is detectable only in PANC−1 cells and it is abolished by SU5402 (**D**,**E**). Equal loading was assessed with anti−actin or tubulin antibodies. Results are expressed as mean value ± SD (*n* = 3). Densitometric analysis was performed as reported in material and methods. ANOVA with Tukey’s multiple comparison test: * *p* ≤ 0.05. Original blots see Figure S4.

We first investigated to what extent the aberrant expression of the mesenchymal FGFR2c isoform in PDAC cell lines could impact on the intracellular signaling activation in response to FGFs. To this aim, we assessed the expression levels of the epithelial and the mesenchymal variants of FGFR2 (FGFR2b and FGFR2c, respectively) in PANC-1 and MiaPaCa-2 pancreatic tumor cell lines, selected for different levels of FGFR2c [10,11], and we compared them with those observed in human keratinocyte HaCaT cell line and normal human fibroblasts (HFs), used as positive controls for FGFR2b and FGFR2c expression, respectively. mRNA levels were assessed by real time RT-PCR and normalized respect to 18SrRNA. Results showed that FGFR2c expression was significantly higher in PANC-1 cells, compared to Mia-PaCa-2 cells (Figure 1A, right panel), while no appreciable levels of FGFR2b mRNA were detected in both PDAC cell lines, compared to HaCaT cells (Figure 1A, left panel). 

Then, in the two selected PDAC cells expressing different levels of FGFR2c, we investigated the activation of the intracellular signaling in response to FGF2, the FGF family member, which does not bind the epithelial FGFR2b, but interacts with other FGFRs, including FGFR2c. Particular attention was paid to MEK/ERK and AKT/MTOR, which are the two main signaling pathways responsible not only for cell growth deregulation and survival, but also for EMT induction [4,5] and for the modulation of autophagy [2] in pancreatic cancer cells. Western blot analysis showed that an enhancement of the basal phosphorylation of ERK1/2 after FGF2 stimulation was higher in PANC-1 respect to Mia PaCa-2 cells (Figure 1B), while that of AKT was exclusively in PANC-1 cells (Figure 1C). The treatment with the FGFR2 kinase inhibitor SU5402 was able to abrogate these effects (Figure 1B,C), confirming their dependence from FGFR2c activation. The higher sensitivity of PANC-1 cells to FGF2 was also evident, downstream AKT, as it increased phosphorylation of MTOR (Figure 1D) and of its substrate S6K (Figure 1E), both events that were abolished by the presence of SU5402 (Figure 1D,E). Therefore, a higher expression of FGFR2c resulted in a more pronounced responsiveness of tumor cells to FGF2 in terms of intracellular signaling activation.

### 3.2. FGFR2c Expression Enhances the EMT Phenotype in Response to FGF2

Then, we shifted our attention to EMT-related gene profile expressed in PDAC cells expressing different levels of FGFR2c. We found that the expression levels of the transcription factors Snail1, FRA1 and STAT3, which we previously identified as involved in FGFR2c-mediated EMT [8,21], overlapped with those of FGFR2c, appearing significantly higher in PANC-1 cells, compared to MiaPaCa-2 cells (Supplementary Figure S1A). Consistent with what was observed for the EMT-related transcription factors, the modulation of epithelial/mesenchymal markers compatible with EMT also appeared to overlap FGFR2c expression, displaying a more pronounced downregulation of the epithelial markers E-cadherin and a higher expression of the mesenchymal marker vimentin in PANC-1 cells compared to Mia PaCa-2 cells (Supplementary Figure S1B). HaCaT cells and the primary culture of human fibroblasts (HFs) were used as positive controls for the expression of epithelial and mesenchymal markers, respectively (Supplementary Figure S1B). Thus, in PDAC cells, the EMT expression profile appears to be related to the extent of FGFR2c expression.

**Figure 2:**
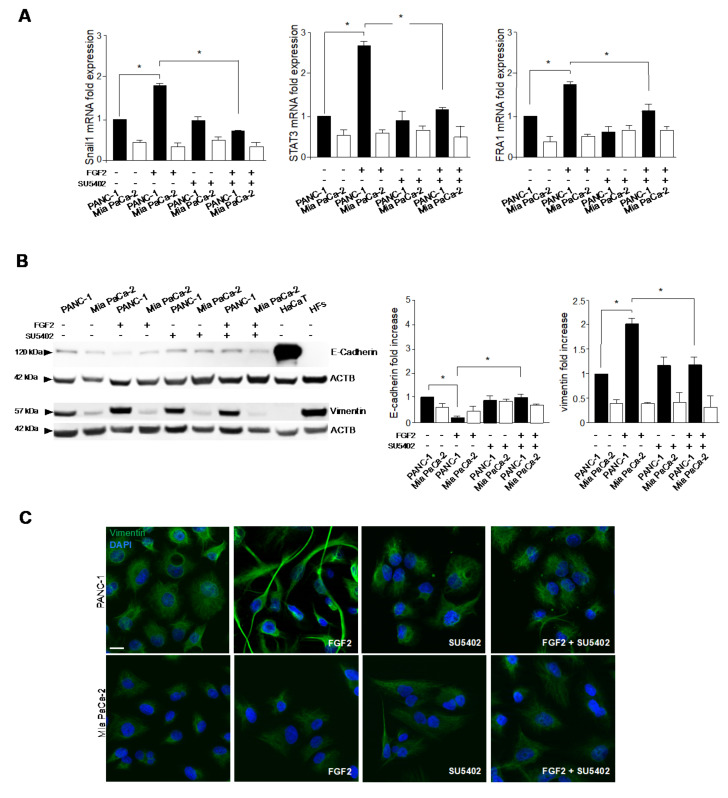
FGFR2c expression impacts on the enhancement of EMT phenotype in response to FGF2. PANC−1 and Mia PaCa−2 cells were left untreated or stimulated with FGF2 in the presence or absence of SU5402, as above. HaCaT cells and HFs were used as controls for the expression of E−cadherin and vimentin, respectively. (**A**) Real−time RT−PCR shows the induction of the EMT−related transcription factors Snail1, STAT3 and FRA1 by FGF2 stimulation only in PANC−1 and its reversion by SU5402. Results are expressed as mean value ± SD (*n* = 3). ANOVA with Tukey’s multiple comparison test: * *p* ≤ 0.05. (**B**) Western blot analysis shows that, only in PANC−1 cells, the very low levels of the epithelial marker E−cadherin and the high levels of the mesenchymal marker vimentin are further decreased and increased, respectively, by FGF2 stimulation. The presence of SU5402 reverses these effects. E−cadherin and vimentin expression are not significantly changed by FGF2 treatment in Mia PaCa−2 cells. Equal loading was assessed with the anti−actin antibody. Results are expressed as mean value ± SD (*n* = 3). The densitometric analysis was performed as reported above. ANOVA with Tukey’s multiple comparison test: * *p* ≤ 0.05. Original blots see Figure S4. (**C**) Immunofluorescence analysis shows that FGF2 stimulation leads PANC−1 cells to detach from each other and to assume a spindle shape, while Mia PaCa−2 morphology remains unchanged. The anti−vimentin immunostaining is increased by the stimulation only in PANC−1 cells. All the effects are abrogated by SU5402. Nuclei were stained with DAPI. Bar: 10 µm.

To assess to what extent the expression level of FGFR2c could impact on the enhancement of EMT features in response to microenvironmental factors, we analyzed the modulation of the EMT-related transcription factors Snail1, FRA1 and STAT3 after FGF2 stimulation. Real time RT-PCR showed that all the three transcription factors were highly induced by growth factor stimulation in PANC-1, but not in MiaPaCa-2 cells (Figure 2A), and this effect was efficiently counteracted by SU5402 (Figure 2A) confirming its dependence on FGFR2 signaling. Biochemical analysis was performed to assess the contribution of FGFR2c expression and signaling on epithelial/mesenchymal marker modulation. The results showed that, only in PANC-1 cells, the very low levels of the epithelial marker E-cadherin and the high levels of the mesenchymal marker vimentin appeared further decreased and increased, respectively, by FGF2 stimulation (Figure 2B). Again, the efficiency of SU5402 in reversing these effects (Figure 2B) confirmed the dependence on FGFR2c activation and signaling. In contrast, the hardly detectable levels of E-cadherin, as well as the lower levels of vimentin observed in Mia PaCa-2 cells compared to PANC-1 cells (Figure 2B), appeared not significantly affected by FGF2 treatment (Figure 2B). Our biochemical findings were also validated by immunofluorescence approaches, which showed how FGF2 stimulation did not substantially impact on Mia PaCa-2 morphology (Figure 2C), while it forced PANC-1 cells to detach from each other and to assume a spindle shape (Figure 2C). In addition, the immunostaining with anti-vimentin appeared significantly increased by FGF2 and abrogate by SU5402 only in PANC-1 cells (Figure 2C). 

**Figure 3:**
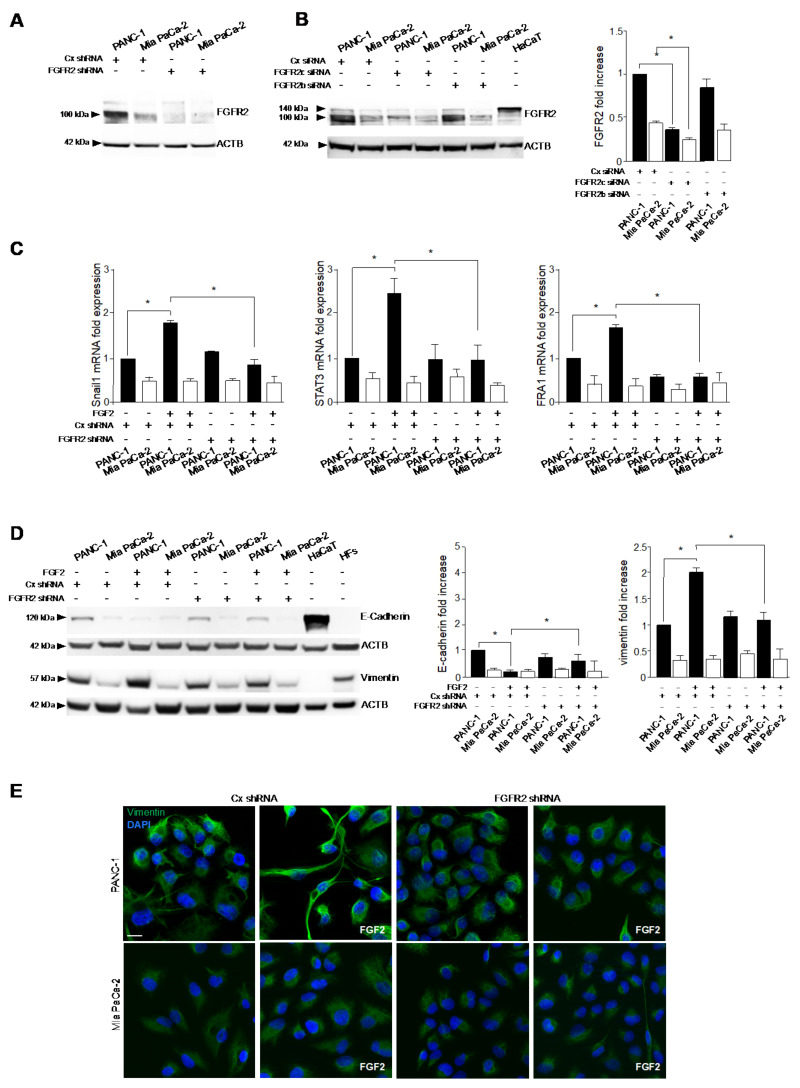
FGFR2c depletion affects the responsiveness of PANC−1 cells to FGF2 in terms of enhancement of the mesenchymal expression profile. PANC−1 and Mia PaCa−2 cells were stably transduced with FGFR2 shRNA or alternatively transfected with specific FGFR2b siRNA or FGFR2c siRNA. Unrelated shRNA (Cx shRNA) or siRNA (Cx siRNA) were used as negative control. Cells were left unstimulated or stimulated with FGF2 as above. HaCaT cells were used as positive control for the expression of FGFR2 and E−cadherin, while HFs for that of vimentin. (**A**) Western blot analysis shows the efficiency of the stable protein depletion of FGFR2 by shRNA transduction. (**B**) Especially in PANC−1 cells, the transfection with FGFR2c siRNA, but not that with FGFR2b siRNA, induces a decrease of FGFR2 band, which is comparable to that obtained using the generic FGFR2 shRNA (shown in A). Equal loading was assessed with the anti−actin antibody. Results are expressed as mean value ± SD (*n* = 3). The densitometric analysis was performed as reported above. ANOVA with Tukey’s multiple comparison test: * *p* ≤ 0.05. (**C**) Real−time RT−PCR shows that FGFR2 depletion abolishes the increase of Snail1, STAT3 and FRA1 induced only in PANC−1 cells by FGF2 stimulation. Results are expressed as mean value ± SD (*n* = 3). ANOVA with Tukey’s multiple comparison test: * *p* ≤ 0.05. (**D**) Western blot analysis shows that the stable depletion of FGFR2 makes PANC−1 unresponsive to FGF2, in terms of further repression of E−cadherin and vimentin enhancement. Equal loading was assessed with the anti−actin antibody. Results are expressed as mean value ± SD (*n* = 3). The densitometric analysis was performed as reported above. ANOVA with Tukey’s multiple comparison test: * *p* ≤ 0.05. (**E**) Immunofluorescence analysis shows that, in PANC-1 cells, the enhancement of vimentin immunostaining and the tendency to assume a spindle−shaped morphology in response to FGF2 are abolished by FGFR2 depletion. Bar: 10 µm. Original blots see Figure S4.

To definitely confirm that the greater responsiveness displayed by PANC-1 cells to FGF2 in terms of acquisition of a more pronounced mesenchymal expression profile and morphology might be ascribed to a higher expression of FGFR2c, we performed a stable protein depletion of FGFR2 by specific short hairpin RNA (FGFR2 shRNA) transfection, whose efficiency was verified by biochemical approaches (Figure 3A). Parallel experiments performed using isoform specific small interfering RNAs showed that, especially in PANC-1 cells, the transfection with FGFR2c siRNA, but not that with FGFR2b siRNA, induced a decrease of the band at the molecular weight of 100kDa (Figure 3B), which was comparable to that obtained using the generic FGFR2 shRNA (Figure 3A). These results confirmed that the transfection with the FGFR2 shRNA actually results in an efficient depletion of the receptor mesenchymal variant. It is worth noting that, as expected [12], PDAC cell lines and HaCaT keratinocytes, used as positive control for FGFR2b expression, expressed two different variants of FGFR2 isoforms, differing for the presence or not of the first Ig loop in the extracellular domain, which implies different molecular weights (100 and 140 kDa, respectively) [22]. Then, analyzing the expression of the EMT-related transcription factors Snail1, STAT3 and FRA1 by real-time RT-PCR, we found that their increase, induced only in PANC-1 by FGF2 stimulation (Figure 3C), was abolished by the receptor depletion (Figure 3C). Comparable results were obtained in biochemical and immunofluorescence experiments, performed to assess the impact of FGFR2c depletion on the modulation of the epithelial/mesenchymal markers and on the changes in cell morphology in response to FGF2. In fact, in line to what was observed in the presence of SU5402, the stable depletion of FGFR2 abolished PANC-1 responsiveness to FGF2, in terms of E-cadherin repression and vimentin enhancement (Figure 3D,E). In addition, these cells lost the tendency to assume a spindle-shaped morphology in response FGF2 stimulation, conserving their cobblestone shape and the ability to growth in packed colonies (Figure 3E). Thus, the different responsiveness of PANC-1 and Mia PaCa-2 cells to paracrine FGFs in terms of acquisition of EMT phenotype appears to be mostly attributable on their different expression of FGFR2c.

### 3.3. The Activation of PKCε Is the Key Molecular Event Downstream FGFR2c Underlying EMT Induction

**Figure 4:**
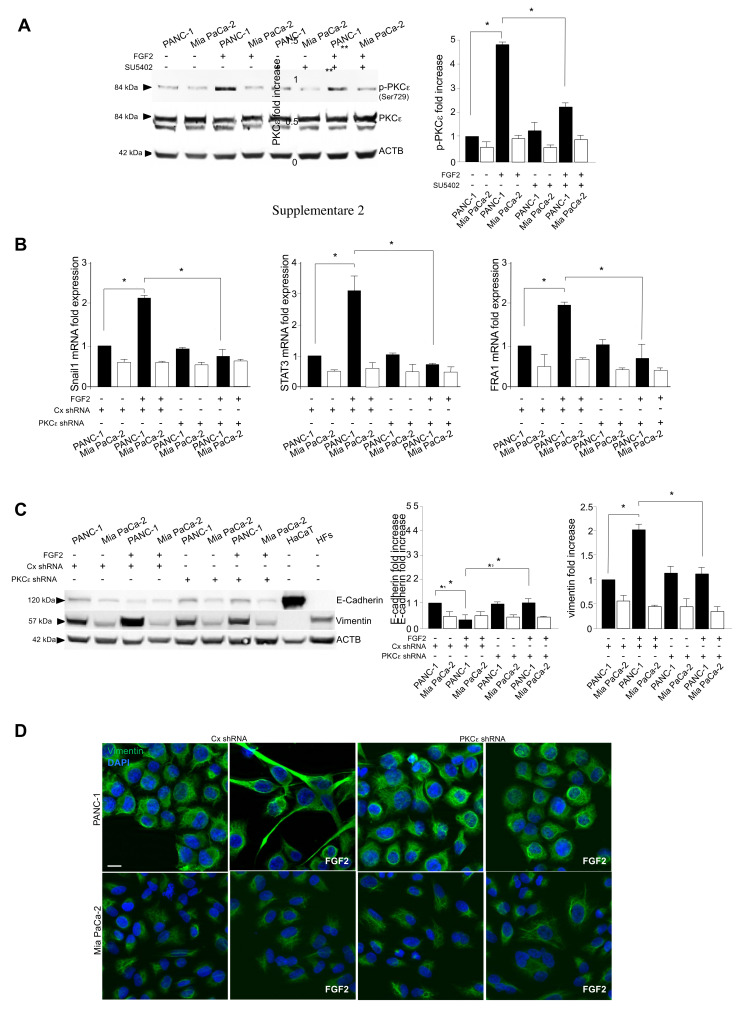
The depletion of PKCε interferes with FGF2−triggered EMT phenotype. PANC−1 and Mia PaCa−2 cells were left untransduced or stably transduced with PKCε shRNA or with an unrelated shRNA, as negative control. Cells were left untreated or stimulated with FGF2 in presence or absence of SU5402 as above. HaCaT cells and HFs were used as positive controls for epithelial/mesenchymal marker expression, as reported above. (**A**) Western blot analysis shows that the increase of phosphorylation of PKCε is observed upon FGF2 stimulation only in PANC−1 cells and this effect is abolished by SU5402. Equal loading was assessed with the anti−actin antibody. Results are expressed as mean value ± SD (*n* = 3). The densitometric analysis was performed as reported above. ANOVA with Tukey’s multiple comparison test: * *p* ≤ 0.05. (**B**) Real−time RT−PCR shows that the induction of Snail1, STAT3 and FRA1 only in PANC−1 cells in response to FGF2 is repressed upon PKCε depletion. Results are expressed as mean value ± SD (*n* = 3). ANOVA with Tukey’s multiple comparison test: * *p* ≤ 0.05. (**C**) Western blot analysis highlights that PKCε knockdown also counteracted the repression of E−cadherin, as well as the upregulation of vimentin induced by FGF2 in PANC−1 cells. Equal loading was assessed with the anti−actin antibody. Results are expressed as mean value ± SD (*n* = 3). The densitometric analysis was performed as reported above. ANOVA with Tukey’s multiple comparison test: * *p* ≤ 0.05. (**D**) Immunofluorescence analysis shows that PKCε silencing interferes with the enhancement of vimentin expression, as well as with the tendency of PANC−1 cells to assume the mesenchymal morphology in response to FGF2. Bar: 10 µm. Original blots see Figure S4.

Since we recently found that PKCε is the main signaling substrate contributing to FGFR2c-mediated induction of EMT in human keratinocytes [8,9], the possible involvement of this signaling substrate also in the context of pancreatic cancer has been investigated in this work. To this aim, the extent of PKCε activation in the selected PDAC cell lines was firstly assayed by analyzing the phosphorylation of its Ser 729 site, which depends on the internal catalytic activity and is a widely recognized indicator of PKCε activation [23,24]. Western blot analysis showed that an appreciable increase of phosphorylation of PKCε at this autophosphorylation site was detected only in PANC-1 cells upon FGF2 stimulation (Figure 4A), which was abolished by SU5402 (Figure 4A), confirming its close dependence on FGFR2c activation. In addition, the absence of a detectable increase of phosphorylation in MiaPaCa-2 cells also suggests that PKCε activation could be dependent on FGFR2c expression levels. On the other hand, differently from what observed in human keratinocytes [8], FGF2 stimulation did not induce appreciable changes in PKCε protein levels (Figure 4A).

Then, we analyzed the role exerted by PKCε in the establishment of EMT phenotype, generating PANC-1 and Mia PaCa-2 cell lines stably depleted for PKCε by transfection with specific shRNA. The efficiency of PKCε gene silencing was confirmed by Western blot analysis (Supplementary Figure S2). Real time RT-PCR showed that the induction of the three EMT-related transcription factors downstream FGFR2c, induced in PANC-1 cells by FGF2 (Figure 4B), was significantly repressed by PKCε depletion (Figure 4B). In addition, biochemical experiments highlighted that PKCε knockdown also counteracted the repression of E-cadherin, as well as the upregulation of vimentin induced by FGF2 in these cells (Figure 4C), confirming the interference with EMT induction. Finally, immunofluorescence approaches showed how PKCε depletion was able to counteract either the enhancement of vimentin expression (Figure 4D) or the morphological changes in favor of the mesenchymal feature displayed by PANC-1 cells in response to FGF2 (Figure 4D). These results indicated that PKCε-mediated signaling downstream FGFR2c significantly contribute to the establishment of receptor-dependent EMT phenotype.

### 3.4. PKCε Signaling Negatively Impacts on the Autophagic Process

**Figure 5:**
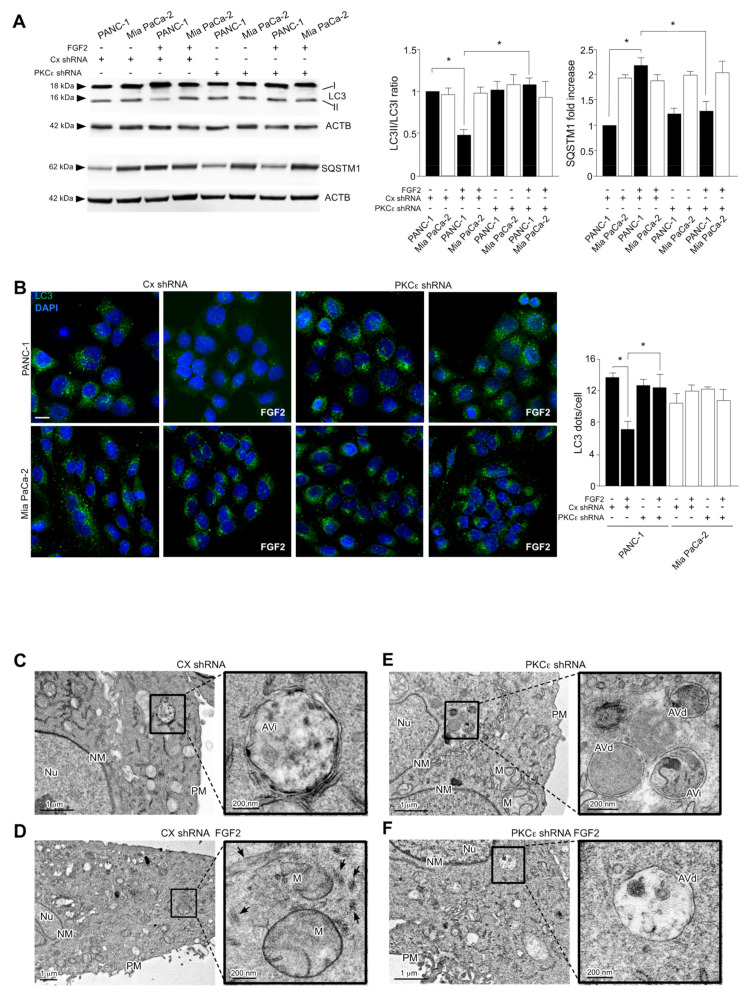
PKCε depletion also negatively impacts on FGF2−dependent inhibition of autophagy. PANC−1 and MiaPaCa−2 cells stably transduced with PKCε shRNA or with an unrelated shRNA were left untreated or stimulated with FGF2 as above. (**A**) Western blot analysis shows that PKCε knockdown abolishes the decrease of the autophagic marker LC3−II, as well as the increase of the autophagic substrate SQSTM1, induced by FGF2 stimulation exclusively in PANC−1 cells. Equal loading was assessed with the anti-actin antibody. Results are expressed as mean value ± SD (*n* = 3). The densitometric analysis was performed as reported above. ANOVA with Tukey’s multiple comparison test: * *p* ≤ 0.05. (**B**) Quantitative immunofluorescence analysis shows that the reduction of LC3 positive dots per cell, evident only in PANC−1 upon FGF2 is reversed by PKCε depletion. Quantitative analysis was performed as described in Materials and Methods, and results are expressed as mean values ± SD (*n* = 3). ANOVA with Tukey’s multiple comparison test: * *p* ≤ 0.05. (**C**–**F**) Ultrastructural analysis by transmission electron microscopy (TEM) shows initial autophagic vacuoles (AVi) with double isolation membrane in the cytoplasm of unstimulated PANC−1 Cx shRNA cells (**C**, magnification box). The examination of PANC−1 Cx shRNA stimulated with FGF2 shows a spindle−like shape, a reduced presence of AVs compared to unstimulated cells, and a higher cytoplasmatic complexity, with several intracellular filaments (**D**), arrows in the magnification box, possibly corresponding to vimentin bundles (**D**). AVi and degradative (AVd) autophagic vacuoles in the cytoplasm of both unstimulated and FGF2−stimulated PKCε shRNA cells (see magnification boxes). AVi: Initial autophagic vacuole; AVd: degradative autophagic vacuole; M: mitochondrion; Nu: nucleus; NM: nuclear membrane; PM: plasma membrane. Bars: 1 µm, 200 nm. Original blots see Figure S4.

We have recently proposed a role of PKCε-mediated signaling not only in FGFR2c-mediated induction of EMT, but also in FGFR2c-dependent inhibition of the autophagic process in human keratinocytes [21]. Therefore, we investigated here the possible contribution of PKCε on autophagy also in the specific context of pancreatic cancer. Western blot analysis showed that PKCε knockdown abolished the decrease of the widely recognized autophagic marker LC3-II, induced by FGF2 stimulation exclusively in PANC-1 cells (Figure 5A). In addition, in these cells, PKCε depletion also counteracted the accumulation of the autophagy substrate SQSTM1 in response to FGF2 (Figure 5A), confirming the efficient reactivation of the autophagic flux. Parallel quantitative immunofluorescence analysis showed that the reduction of LC3 positive dots per cell, evident only in PANC-1 cultures stimulated with FGF2 (Figure 5B), was efficiently reversed by the stable depletion of PKCε (Figure 5B). Comparable results were obtained counteracting FGFR2c signaling and expression by SU5402 or FGFR2 shRNA transfection, respectively (Supplementary Figure S3A,B), demonstrating that the negative effects on autophagy exerted by PKCε upstream requires FGFR2c activation.

The role played by PKCε in the repression of autophagy was further confirmed by electron microscopy studies, performed in PANC-1 cells stably transfected with PKCε shRNA or with control shRNA (Cx shRNA). Ultrastructural examination, performed by transmission electron microscopy (TEM), revealed that the reduction of autophagic vacuoles, triggered by FGF2 stimulation in control cells (Figure 5C,D) was counteracted by PKCε depletion, which enabled cells to maintain a higher number of autophagic structures in the cytoplasm also after FGF2 stimulation (Figure 5E). In addition, PANC-1 Cx shRNA cells, but not PANC-1 PKCε shRNA cells, appeared elongated in response to FGF2 treatment and their cytoplasm resulted enriched in vimentin filament bundles (Figure 5C, arrows). These ultrastructural observations are consistent with our immunofluorescence data (see Figure 4D) and confirm the ability of PKCε knockdown in reversing FGF2-induced mesenchymal phenotype. Thus, in agreement with our previous observations in human keratinocytes [8,9], at least in PANC-1 cells, PKCε-mediated signaling activated downstream FGFR2c appears not only to be involved in EMT induction, but also to exert a not negligible inhibitory effect on autophagy.

### 3.5. PKCε Signaling Interferes with Autophagy Converging on ERK1/2 Pathway

**Figure 6:**
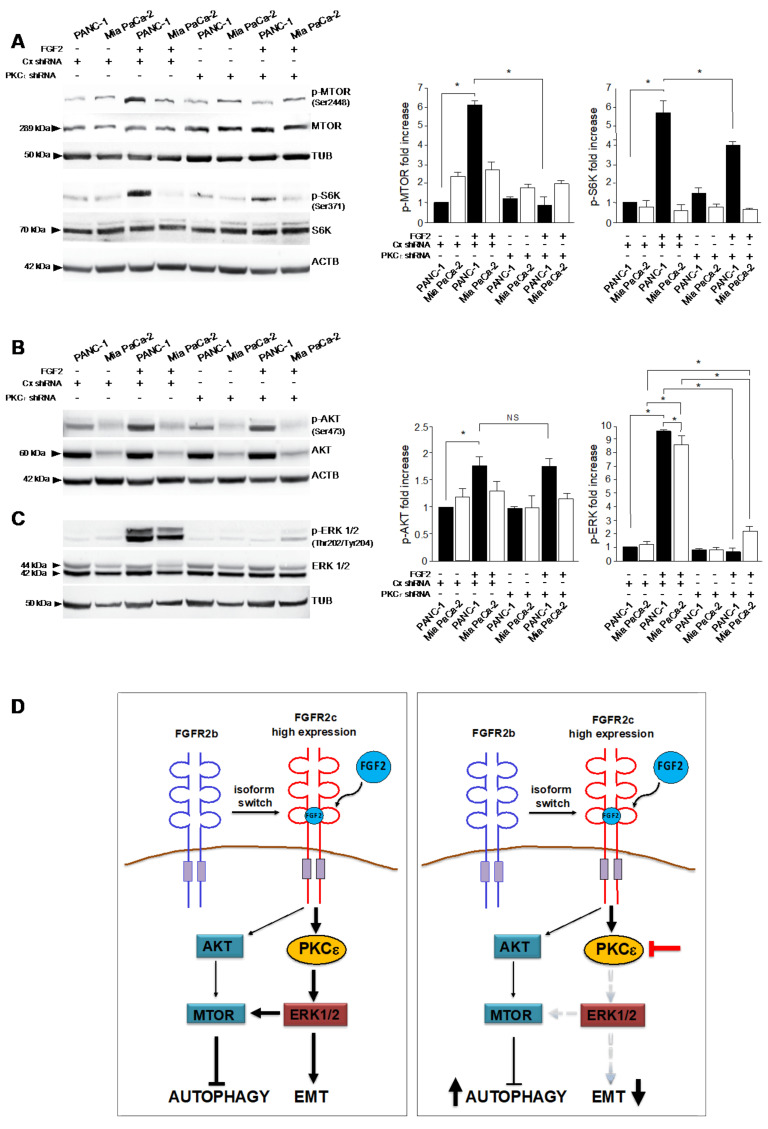
PKCε signaling shut−off by PKCε protein depletion interferes with both MTOR and ERK1/2 signaling pathways. PANC−1 and Mia PaCa−2 cells stably transduced with PKCε shRNA or with an unrelated shRNA were left untreated or stimulated with FGF2 as above. (**A**) Western blot analysis shows that the increase of phosphorylation of MTOR and S6K, evident after FGF2 stimulation only in PANC−1 cells, are strongly dampened by PKCε knockdown. (**B**) No corresponding effects are observed on the AKT phosphorylation. (**C**) The increase of ERK1/2 phosphorylation in response to FGF2, visible in both pancreatic cell lines, is significantly greater in PANC−1 cells and it is reduced in Mia PaCa−2 and completely abolished in PANC−1 by PKCε depletion. Equal loading was assessed with tubulin and anti-actin antibodies. Results are expressed as mean value ± SD (*n* = 3). The densitometric analysis was performed as reported above. ANOVA with Tukey’s multiple comparison test: * *p* ≤ 0.05. (**D**) Schematic drawing representing the role of PKCε as key hub signaling molecule downstream FGFR2c, whose activation simultaneously counteracts autophagy and drives EMT bypassing AKT and directly converging on ERK1/2. PKCε knockdown results in a simultaneous reversion of these effects. Original blots see Figure S4.

To clarify the molecular mechanisms underlying the involvement of PKCε in the autophagic process, we focused our attention on MTOR, which is considered the main negative regulator of autophagy also in pancreatic cancer cells [2,14]. Western blot analysis revealed that the phosphorylation of MTOR, as well as that of its substrate S6K, evident after FGF2 stimulation particularly in PANC-1 cells (Figure 6A), were strongly dampened by PKCε knockdown (Figure 6A). Surprisingly, no corresponding effects were observed on the AKT phosphorylation (Figure 6B). Since AKT is the upstream substrate generally responsible for MTOR activation, our unexpected results indicated that PKCε might activate MTOR through an alternative pathway. This possibility appears to be consistent with the peculiar ability, previously described for PKCε in other cellular contexts, to converge on MTOR through the activation of Raf/MEK/ERK signaling [25]. Actually, the important contribution of ERK1/2 signaling in MTOR activation and consequent autophagy inhibition has been widely described in pancreatic cancer cells [2]. Based on these assumptions, we investigated the impact of PKCε signaling on ERK1/2 pathway. Biochemical analysis showed that, in consequence of PKCε depletion, the increase of ERK1/2 phosphorylation in response to FGF2, visible in both pancreatic cell lines (Figure 6C), was reduced in Mia PaCa-2, which maintained a significant residual ERK phosphorylation (Figure 6C), but completely abolished in PANC-1 (Figure 6C). These results indicate that the different expression of FGFR2c displayed by the two PDAC cell lines impact on the dependence on PKCε of ERK1/2 signaling. It is also worth noting that shFGFR2c transduced MiaPaCa-2 cells displayed a higher responsiveness to FGF2 in terms of ERK1/2 phosphorylation compared to non-transduced ones (see Figure 1B in comparison with Figure 6C), even if this phosphorylation remains significantly lower than that shown by PANC-1 cells. This variability of MiaPaCa-2 cell response to FGF2 might be the consequence of different culture conditions. These results indicated that, only in PANC-1 cells, the activation of ERK1/2 pathway upstream depends on PKCε activation. Since ERK1/2 is also a well-known pathway involved in EMT of PDAC cells [4], our results suggest the possibility that, in this tumor context, PKCε signaling, when activated in consequence of highly expression of FGFR2c, could simultaneously repress autophagy and orchestrate the EMT program directly converging on ERK1/2 pathway. 

## 4. Discussion

PDAC is an aggressive tumor whose KRAS constitutive activation is the main hallmark for malignancy [2]. However, since in specific conditions KRAS could be dispensable [26,27], research efforts have been recently focused on the identification of new signaling molecules and pathways, acting bypassing RAS, whose inhibition might significantly impact on PDAC cell malignant phenotype.

FGFR2 isoform switch is an additional oncogenic event occurring during pancreatic carcinogenesis, whose contribution in EMT induction and cell invasion still appears controversial [10,11,12]. Therefore, with the aim to further clarify this topic we took advantage of the use of two PDAC cell lines (PANC-1 and Mia PaCa-2 cells) expressing undetectable levels of the epithelial FGFR2b isoform and different levels of the mesenchymal FGFR2c variant. Performing a detailed biochemical analysis in these cells, we highlighted a responsiveness to FGF2 in terms of AKT/MTOR and ERK1/2 signaling activation whose modulation appeared closely dependent on FGFR2c expression levels and on receptor activation, as demonstrated by its abolishment by the FGFR2 kinase inhibitor SU5402. 

Then, focusing on the impact on EMT signature, we found that PANC-1 cells, which express higher levels of FGFR2c compared to Mia PaCa-2 cells, displayed higher expression of the EMT-related transcription factors, as well as a more pronounced modulation of epithelial and mesenchymal markers compatible with a pathological EMT. In addition, a clear enhancement of this EMT expression profile after FGF2 stimulation, as well as the acquisition of a mesenchymal morphology in response to FGF2, occurred exclusively in PANC-1 cells and were counteracted by FGFR2c kinase activity shut-off or depletion by specific shRNA, confirming their dependence on receptor expression and signaling. These results may suggest that, in the in vivo cancer context, the extent of FGFR2c aberrant expression could heavily affect tumor cell responsiveness to paracrine factors released by microenvironmental cells, such as cancer associated fibroblasts (CAFs). This higher sensitivity could result in an intense activation of intracellular signaling and consequent enhancement of malignant features. Our findings are in line with previous studies, pointing on the relevance of CAFs and CAF-released factors, such as FGF2, in establishing a more aggressive behaviors in pancreatic cancer cells [28,29]. 

We have also been interested in the signaling pathways and substrates of downstream FGFR2c possibly responsible for the establishment of an EMT-related phenotype, paying particular attention to PKCε, whose oncogenic role in epithelial cells has been widely described [7]. The choice of PKCε also stems from our recent findings indicating that the activation of this signaling substrate is the key event underlying FGFR2c-mediated EMT in the context of human keratinocytes [8,21]. The involvement of PKCε was investigated taking advantage of the use of specific shRNA approaches, which showed that PKCε depletion strongly impairs the increase of EMT signature, as well as the morphological changes triggered by FGF2 in PANC-1 cells. Interestingly, only in these cells PKCε phosphorylation/activation is appreciable, suggesting that PKCε activation could be dependent on FGFR2c expression rate. Since PKCs are considered “RAS-independent” signaling substrates activated by several membrane receptors, including FGFRs [6], the identification of one of PKC family members as a pivotal signaling effector in the establishment of EMT phenotype (and possibly a higher aggressive behavior) could represent a fundamental advance towards new therapeutic strategies aimed to bypass the “undruggable” target RAS. Interestingly, we also found that PKCε silencing abolished the ability of FGF2 to repress autophagy, another important process contributing to PDAC development and progression [2,14,15]. Autophagy and EMT in cancer are linked in a complex crosstalk [13], which we have recently proposed to be regulated by FGFR2c and, to some extent, by its downstream PKCε-mediated signaling, at least during the early steps of human epidermal carcinogenesis [8,21,30]. In line with our previous data, here we highlighted a negative impact of PKCε downstream FGFR2c on autophagy at least in the PANC-1 cell model, which highly expresses the receptor. However, while autophagy is possibly repressed during the early phases of tumorigenesis, in advanced and aggressive cancers, such as those from which PANC-1 and Mia PaCa-2 cell lines are derived, this process is enhanced, and it is widely described as an oncogenic event sustaining cell survival and metabolism [15]. Similarly to what has been already proposed in PDAC for MEK/ERK signaling in PDAC [14], our findings can be explained considering that a negative regulation of autophagy (such as that exerted by FGR2c and by its PKCε downstream signaling) actually results in an oncogenic effect, as it can counteract tumor cell dependence on autophagy for survival. In this perspective, the specific repression of PKCε not only induces a reversion of EMT, but also increases autophagy, enhancing tumor cell dependence on this survival strategy and consequently their susceptibility to autophagic inhibitors.

In addition, investigating in detail the molecular mechanisms underlying the inhibitory effect exerted by PKCε on autophagy, we found that the depletion of PKCε repressed the phosphorylation/activation of the autophagic inhibitor MTOR, visible only in PANC-1 cells in response to FGF2. These results indicated that, as recently proposed in breast cancer [18], PKCε could repress autophagy activating the canonical MTOR autophagy-related pathway also in PDAC. Moreover, PKCε depletion strongly repressed ERK1/2 phosphorylation in both PDAC cell lines, even if MiaPaCa-2 cells appear to maintain a residual ERK1/2 phosphorylation, suggesting that the dependence of ERK1/2 signaling on PKCε activation is consequent on FGFR2c expression levels. In addition, PKCε depletion appeared ineffective on the phosphorylation of AKT, which is the canonical activator of MTOR, suggesting that, as previously proposed for cardiomyocytes [25], PKCε could bypass AKT and directly activate MTOR via ERK1/2. Considering that ERK1/2 is also a well-known pathway regulating EMT in PDAC [4,5], based on our results, we can speculate that PKCε could represent a key hub signaling molecule downstream highly expressed FGFR2c, whose activation could contribute to simultaneously counteract autophagy and drive EMT bypassing AKT and directly converging on ERK1/2 (see schematic draw, Figure 6D). 

In this promising scenario, further investigations will be required to assess the effectiveness of PKCε specific inhibition, used alone or in combination with FGFR, ERK1/2 and autophagy inhibitors, as advanced therapeutic approaches to prevent and/or reverse tumor aggressive phenotypes. 

## 5. Conclusions

Overall, the results show that FGFR2c expression impacts on cell sensitivity to FGF2 in terms of EMT signature and that PKCε is involved in both receptor mediated EMT response and inhibition of autophagy, pointing on this substrate as a potential therapeutic target in counteracting cancer progression.

## Data Availability

All the data is provided in the article.

## Supplementary Materials

The following are available online at https://www.mdpi.com/article/10.3390/cancers13194993/s1, Figure S1: EMT-related expression profile in PDAC cells expressing different levels of FGFR2c, Figure S2: Efficiency of PKCε depletion by specific shRNA, Figure S3: Impact of FGFR2 shut-off or depletion on the autophagic process, Figure S4: Original western blots
