# Supplementary material for: Role of FGFR2c and Its PKCε Downstream Signaling in the Control of EMT and Autophagy in Pancreatic Ductal Adenocarcinoma Cells
Source: Cancers (Basel). 2021 Oct 5;13(19):4993. doi: 10.3390/cancers13194993 (PMC8508074; doi:10.3390/cancers13194993)
Supplement: Supplementary file 1 [file cancers-13-04993-s001.zip › cancers-1371721-supplementary for proof/Supplementary Figure1 REV2.pdf]

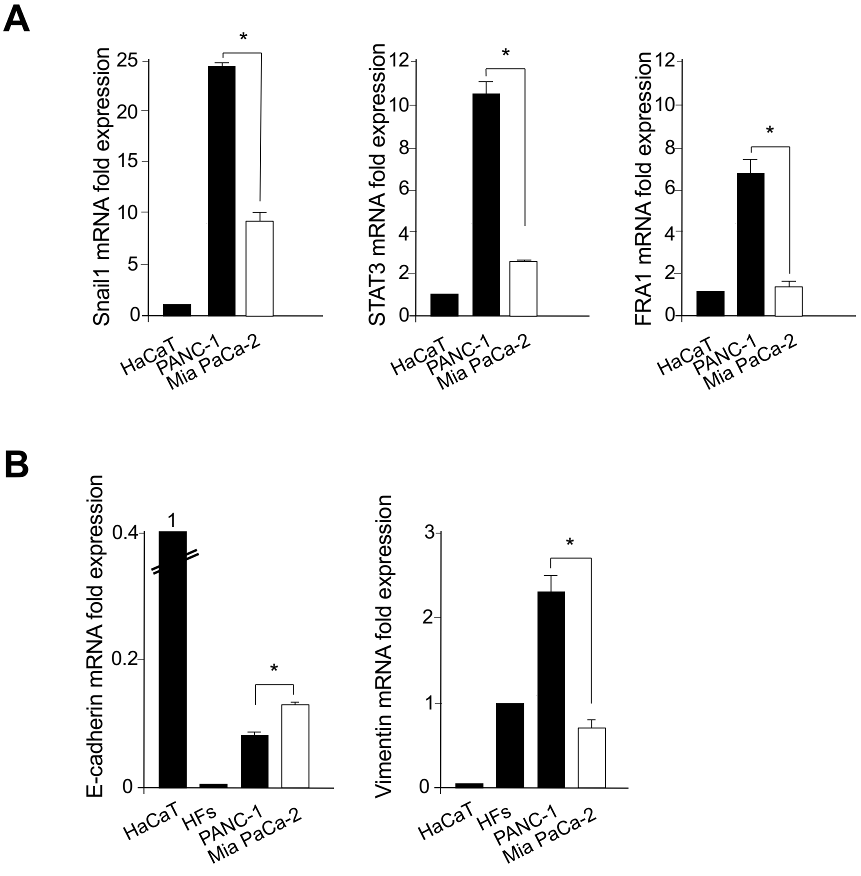

Supplementary Figure 1

EMT-related expression profile in PDAC cells expressing different levels of FGFR2c. (A) Real-time RT-PCR shows that the expression levels of the EMT-related transcription factors Snail1, FRA1 and STAT3 are significantly higher in PANC-1 cells, compared to MiaPaCa-2 cells (B) The expression of the epithelial markers E-cadherin is lower, and that of the mesenchymal marker vimentin is higher in PANC-1 cells compared to Mia PaCa-2 cells. HaCaT cells and primary culture of human fibroblasts (HFs) are used as positive controls for the expression of epithelial and mesenchymal markers, respectivelly. Results are expressed as mean value ± SD (n=3). ANOVA with Tukey's multiple comparison test: * $p < 0.05$.
