# Supplementary material for: Role of FGFR2c and Its PKCε Downstream Signaling in the Control of EMT and Autophagy in Pancreatic Ductal Adenocarcinoma Cells
Source: Cancers (Basel). 2021 Oct 5;13(19):4993. doi: 10.3390/cancers13194993 (PMC8508074; doi:10.3390/cancers13194993)
Supplement: Supplementary file 1 [file cancers-13-04993-s001.zip › cancers-1371721-supplementary for proof/Supplementary Figure2 REV2.pdf]

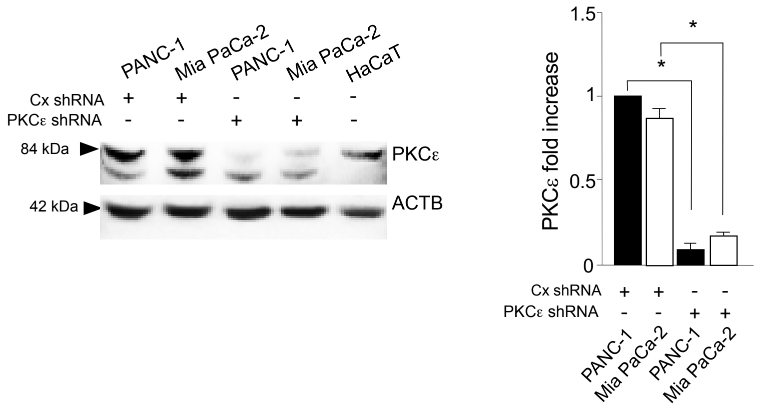

Supplementary Figure S2

Efficiency of PKCε depletion by specific shRNA. PANC-1 and MiaPaCa-2 cells were stably transduced with PKCε shRNA or with an unrelated shRNA, as negative control. HaCaT cells were used as positive control for PKCε expression. Western blot analysis shows the efficiency of PKCε gene silencing. Equal loading was assessed with the anti-actin antibody. Results are expressed as mean value ± SD. The densitometric analysis was performed as reported in materials and methods. ANOVA with Tukey's multiple comparison test: * $p < 0.05$.
