# Supplementary material for: Role of FGFR2c and Its PKCε Downstream Signaling in the Control of EMT and Autophagy in Pancreatic Ductal Adenocarcinoma Cells
Source: Cancers (Basel). 2021 Oct 5;13(19):4993. doi: 10.3390/cancers13194993 (PMC8508074; doi:10.3390/cancers13194993)
Supplement: Supplementary file 1 [file cancers-13-04993-s001.zip › cancers-1371721-supplementary for proof/Supplementary Figure3 REV2.pdf]

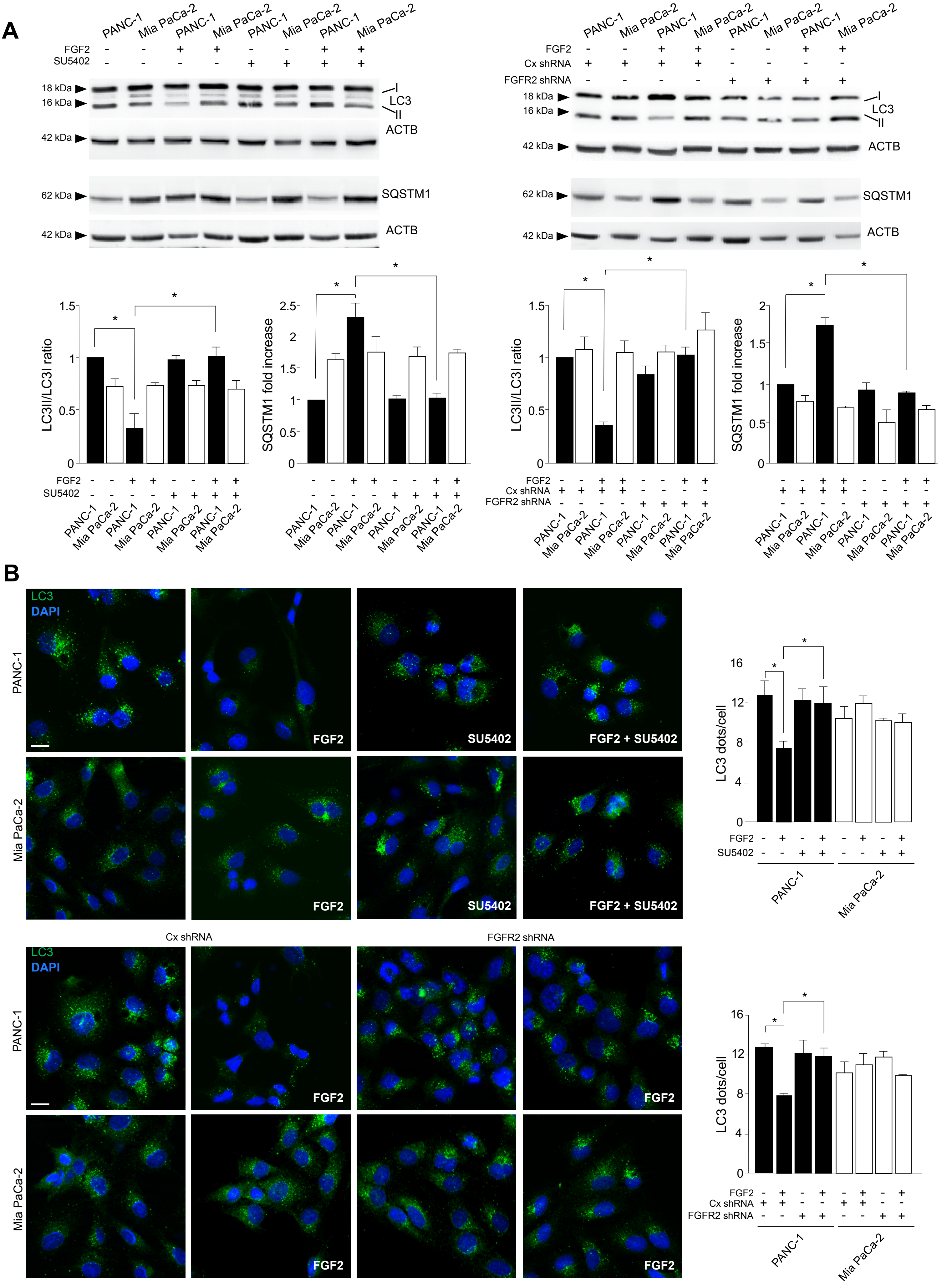

Supplementary Figure 3

Supplementary Figure S3

Impact of FGFR2 shut-off or depletion on the autophagic process. PANC-1 and MiaPaCa-2 cells were left untreated or stimulated with FGF2 in the presence or absence of the FGFR2 inhibitor SU5402. Alternatively, cells were stably transduced with FGFR2 shRNA or with an unrelated shRNA, as negative control and then stimulated with FGF2 as above. (A) Western blot analysis shows that both the presence of SU5402 (left) and FGFR2 protein depletion by shRNA (right) abolish either the decrease of the autophagic marker LC3-II and the accumulation of the autophagy substrate SQSTM1 induced by FGF2 stimulation exclusively in PANC-1 cells. Equal loading was assessed with the anti-actin antibody. Results are expressed as mean value ± SD. The densitometric analysis was performed as reported in materials and methods. ANOVA with Tukey's multiple comparison test: * $p < 0.05$. (B) Quantitative immunofluorescence analysis shows that the reduction of LC3 positive dots per cell, evident only in PANC-1 upon FGF2 is reversed by FGFR2 signaling shut-off (by SU5402, top panel) or depletion (by FGFR2shRNA, bottom panel). Quantitative analysis was performed as described in Materials and Methods, and results are expressed as mean values ± SD. ANOVA with Tukey's multiple comparison test: * $p < 0.05$.
