# Supplementary figures and images for: Role of FGFR2c and Its PKCε Downstream Signaling in the Control of EMT and Autophagy in Pancreatic Ductal Adenocarcinoma Cells
Source: Cancers (Basel). 2021 Oct 5;13(19):4993. doi: 10.3390/cancers13194993 (PMC8508074; doi:10.3390/cancers13194993)

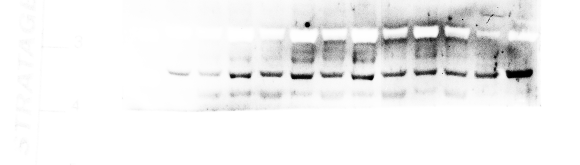

Supplement: Supplementary file 1 [file cancers-13-04993-s001.zip › cancers-1371721-supplementary for proof/cancers-1371721-original-images/Fig. 1/Fig. 1 C ACTB.tif]

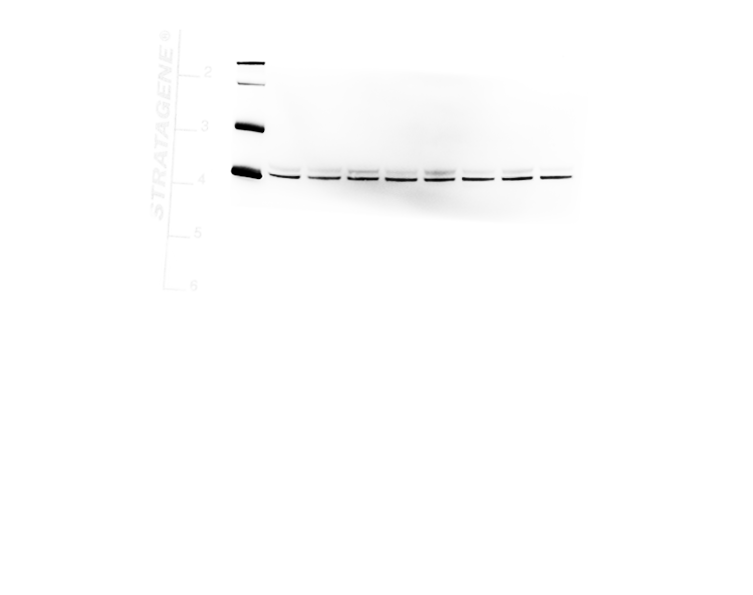

Supplement: Supplementary file 1 [file cancers-13-04993-s001.zip › cancers-1371721-supplementary for proof/cancers-1371721-original-images/Fig. 1/Fig.1 B ERK Tot .tif]

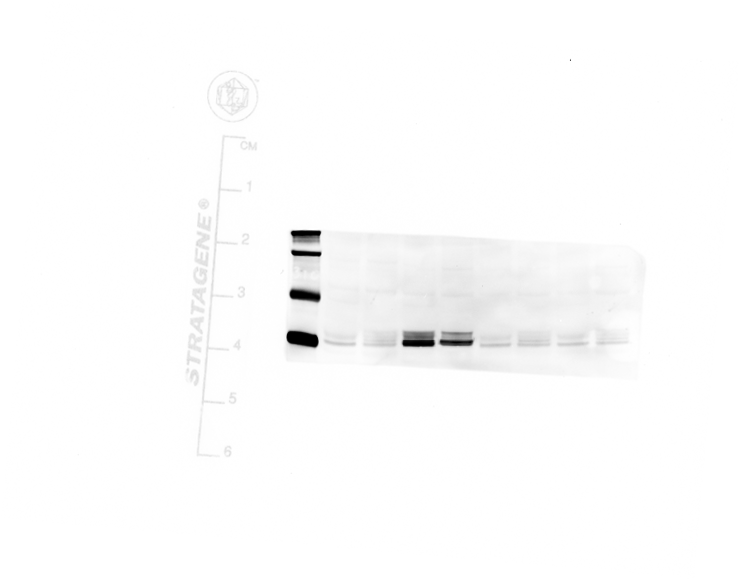

Supplement: Supplementary file 1 [file cancers-13-04993-s001.zip › cancers-1371721-supplementary for proof/cancers-1371721-original-images/Fig. 1/Fig.1 B P-ERK.tif]

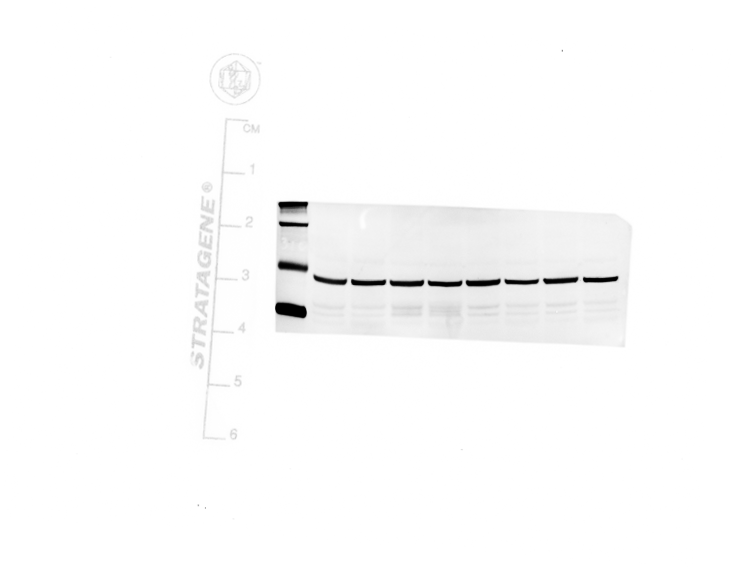

Supplement: Supplementary file 1 [file cancers-13-04993-s001.zip › cancers-1371721-supplementary for proof/cancers-1371721-original-images/Fig. 1/Fig.1 B TUB.tif]

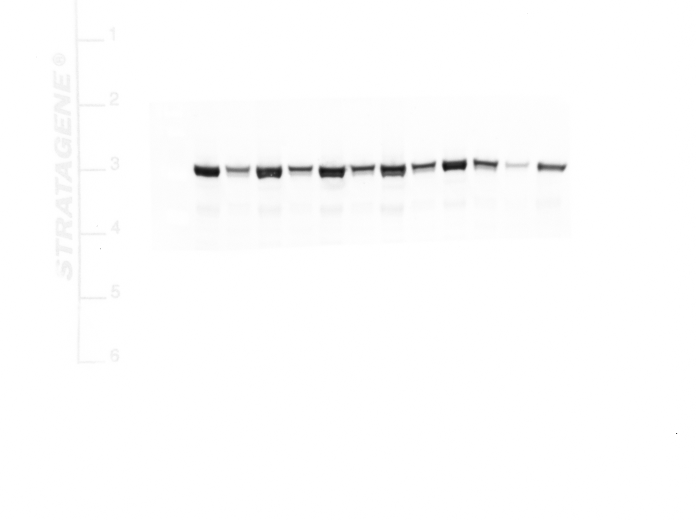

Supplement: Supplementary file 1 [file cancers-13-04993-s001.zip › cancers-1371721-supplementary for proof/cancers-1371721-original-images/Fig. 1/Fig.1 C AKT Tot.tif]

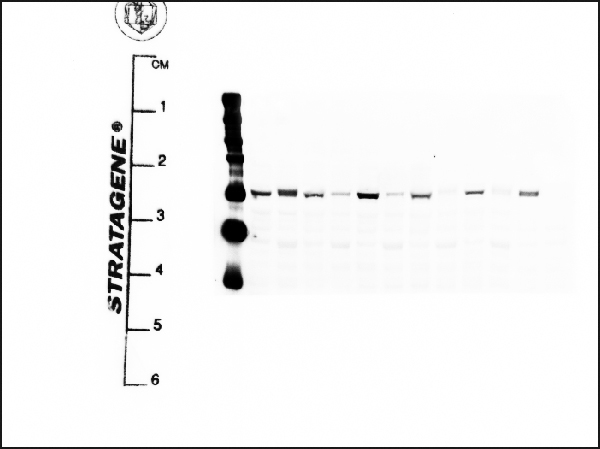

Supplement: Supplementary file 1 [file cancers-13-04993-s001.zip › cancers-1371721-supplementary for proof/cancers-1371721-original-images/Fig. 1/Fig.1 C p-AKT .jpg]

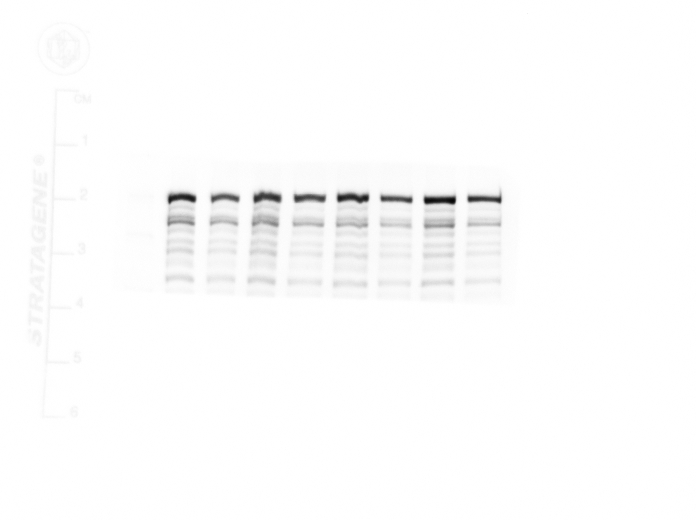

Supplement: Supplementary file 1 [file cancers-13-04993-s001.zip › cancers-1371721-supplementary for proof/cancers-1371721-original-images/Fig. 1/Fig.1 D MTOR.tif]

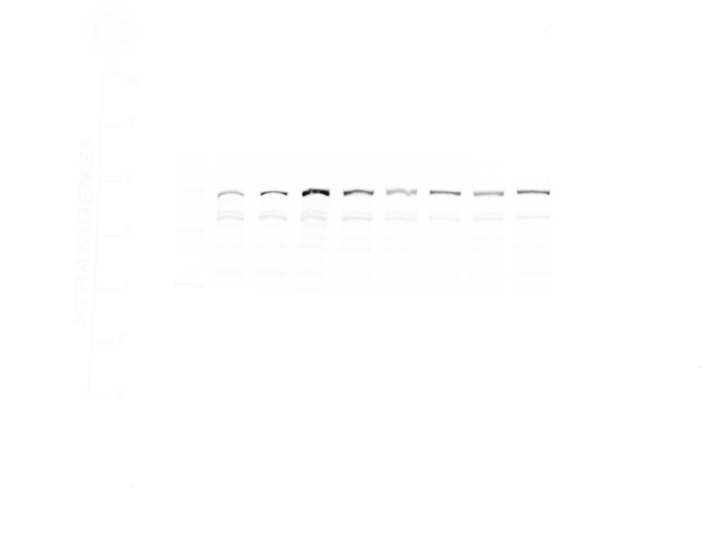

Supplement: Supplementary file 1 [file cancers-13-04993-s001.zip › cancers-1371721-supplementary for proof/cancers-1371721-original-images/Fig. 1/Fig.1 D P-MTOR.tif]

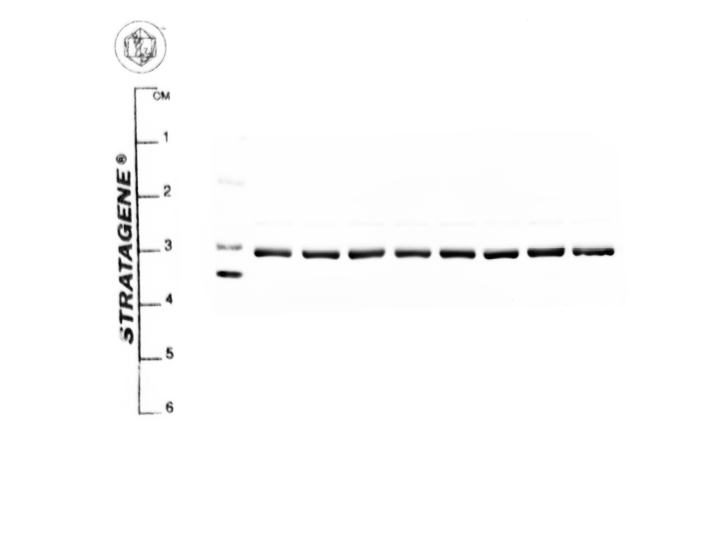

Supplement: Supplementary file 1 [file cancers-13-04993-s001.zip › cancers-1371721-supplementary for proof/cancers-1371721-original-images/Fig. 1/Fig.1 D TUB.tif]

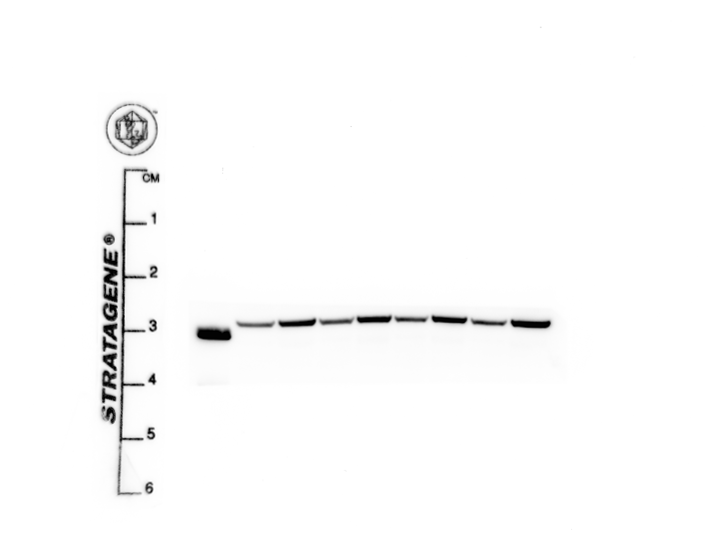

Supplement: Supplementary file 1 [file cancers-13-04993-s001.zip › cancers-1371721-supplementary for proof/cancers-1371721-original-images/Fig. 1/Fig.1 E ACT.tif]

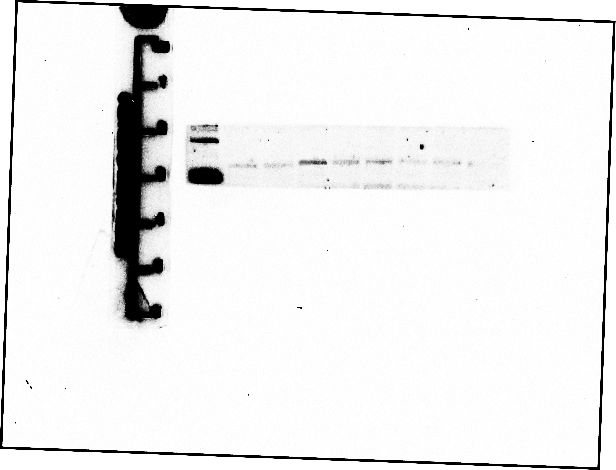

Supplement: Supplementary file 1 [file cancers-13-04993-s001.zip › cancers-1371721-supplementary for proof/cancers-1371721-original-images/Fig. 1/Fig.1 E p-S6K.tif]

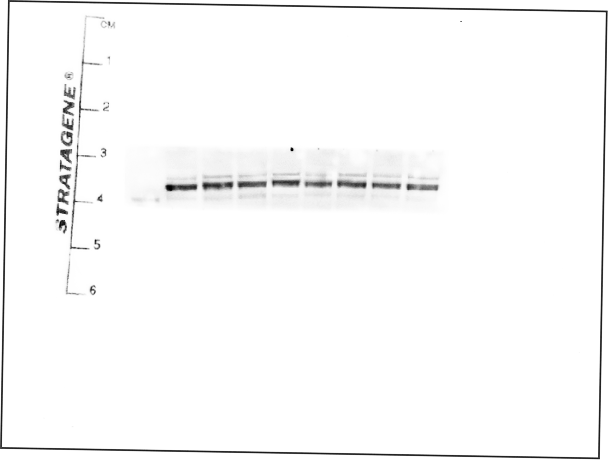

Supplement: Supplementary file 1 [file cancers-13-04993-s001.zip › cancers-1371721-supplementary for proof/cancers-1371721-original-images/Fig. 1/Fig.1 E S6K Tot.tif]

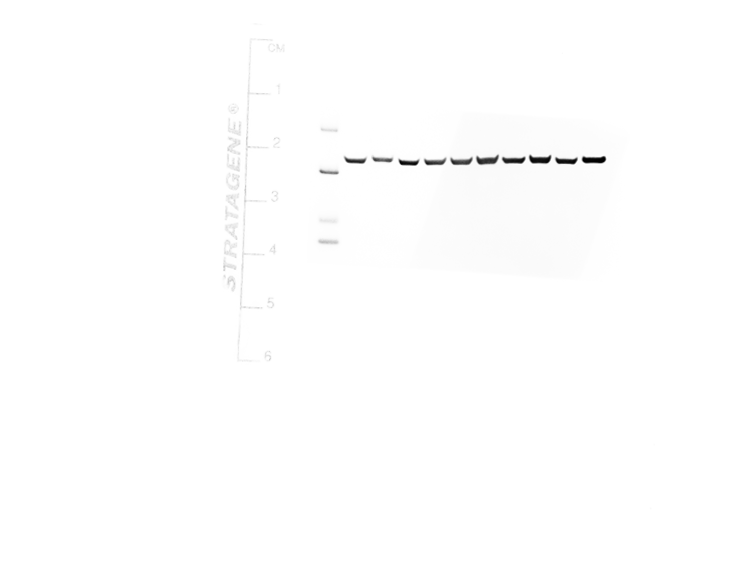

Supplement: Supplementary file 1 [file cancers-13-04993-s001.zip › cancers-1371721-supplementary for proof/cancers-1371721-original-images/Fig. 2/Fig.2 B ACT (ECAD).tif]

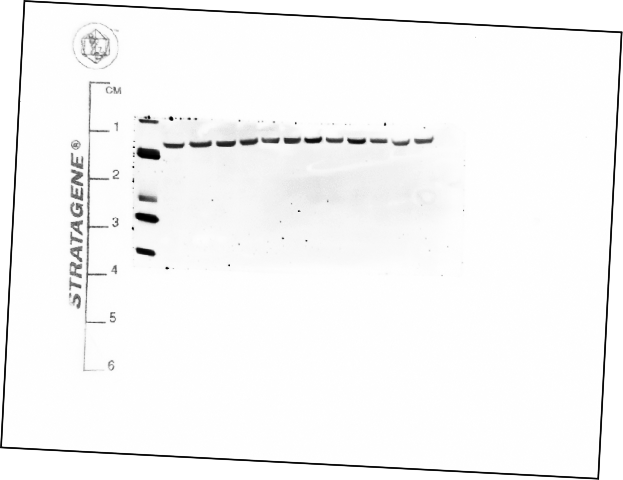

Supplement: Supplementary file 1 [file cancers-13-04993-s001.zip › cancers-1371721-supplementary for proof/cancers-1371721-original-images/Fig. 2/Fig.2 B ACT (Vim).tif]

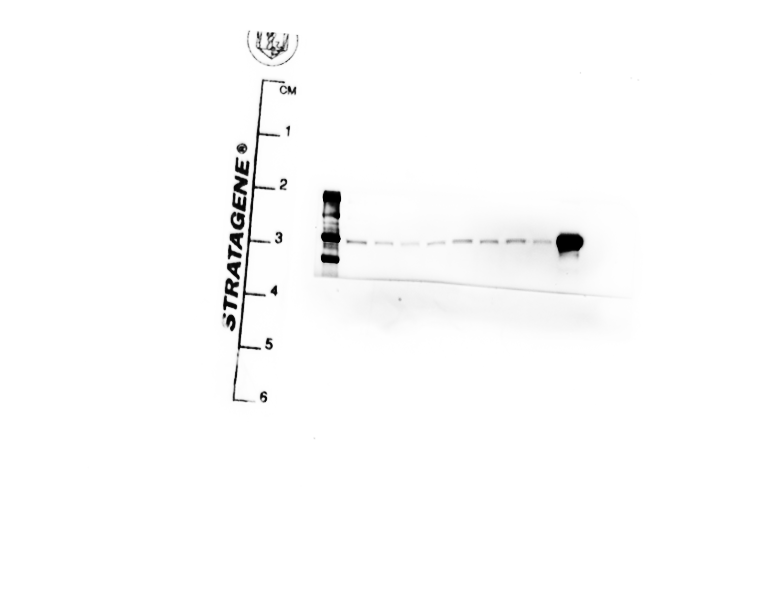

Supplement: Supplementary file 1 [file cancers-13-04993-s001.zip › cancers-1371721-supplementary for proof/cancers-1371721-original-images/Fig. 2/Fig.2 B ECAD.tif]

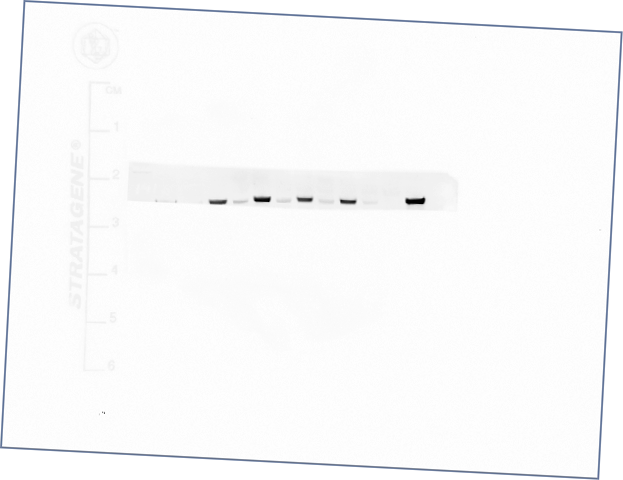

Supplement: Supplementary file 1 [file cancers-13-04993-s001.zip › cancers-1371721-supplementary for proof/cancers-1371721-original-images/Fig. 2/Fig.2 B Vim.tif]

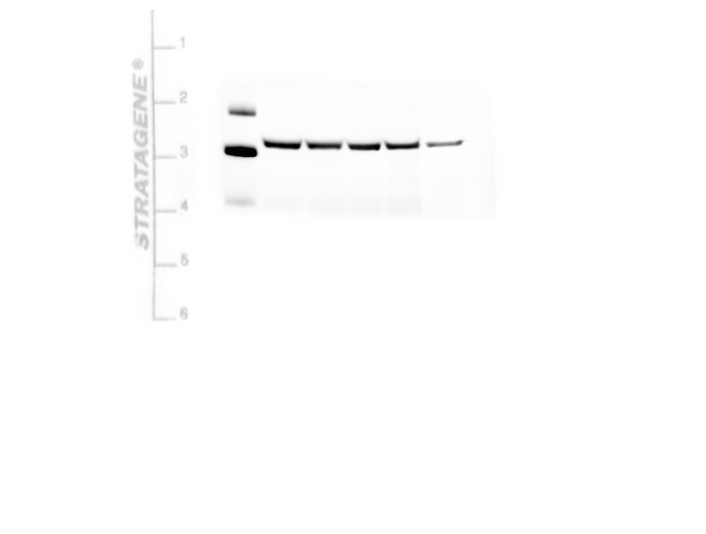

Supplement: Supplementary file 1 [file cancers-13-04993-s001.zip › cancers-1371721-supplementary for proof/cancers-1371721-original-images/Fig. 3/Fig 3 A ACT.tif]

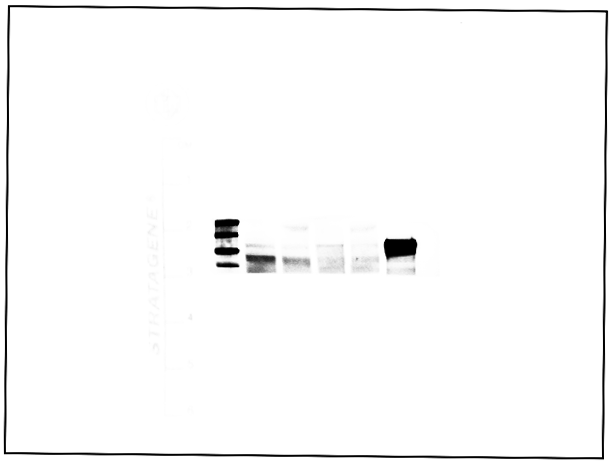

Supplement: Supplementary file 1 [file cancers-13-04993-s001.zip › cancers-1371721-supplementary for proof/cancers-1371721-original-images/Fig. 3/Fig 3 A FGFR2.tif]

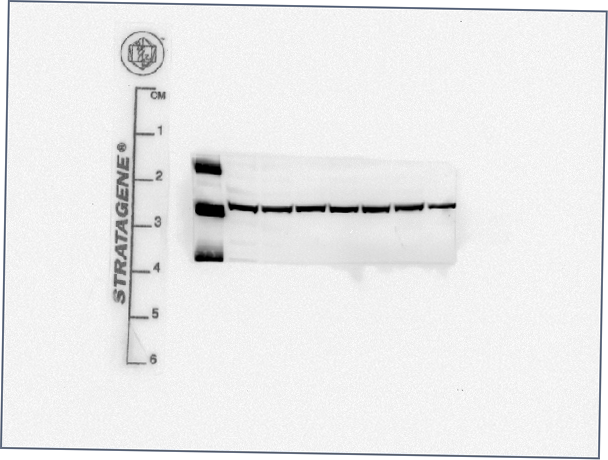

Supplement: Supplementary file 1 [file cancers-13-04993-s001.zip › cancers-1371721-supplementary for proof/cancers-1371721-original-images/Fig. 3/Fig 3 B ACT.tif]

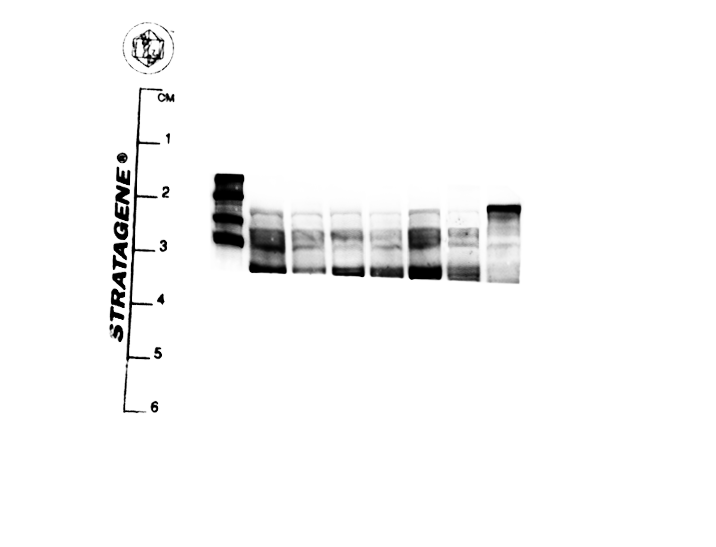

Supplement: Supplementary file 1 [file cancers-13-04993-s001.zip › cancers-1371721-supplementary for proof/cancers-1371721-original-images/Fig. 3/Fig 3 B FGFR2.tif]

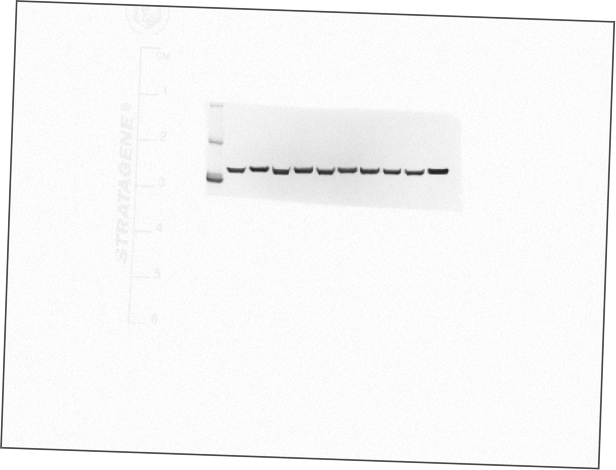

Supplement: Supplementary file 1 [file cancers-13-04993-s001.zip › cancers-1371721-supplementary for proof/cancers-1371721-original-images/Fig. 3/Fig 3 D ACT (ECAD).tif]

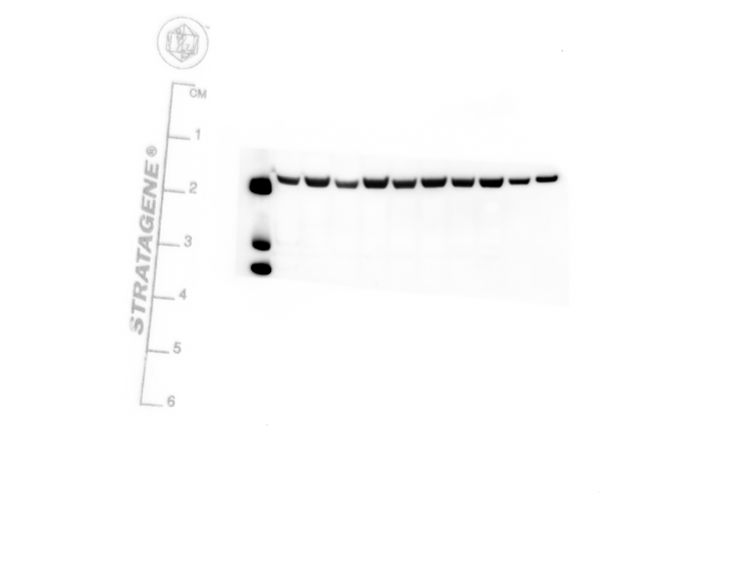

Supplement: Supplementary file 1 [file cancers-13-04993-s001.zip › cancers-1371721-supplementary for proof/cancers-1371721-original-images/Fig. 3/Fig 3 D ACT (Vim).tif]

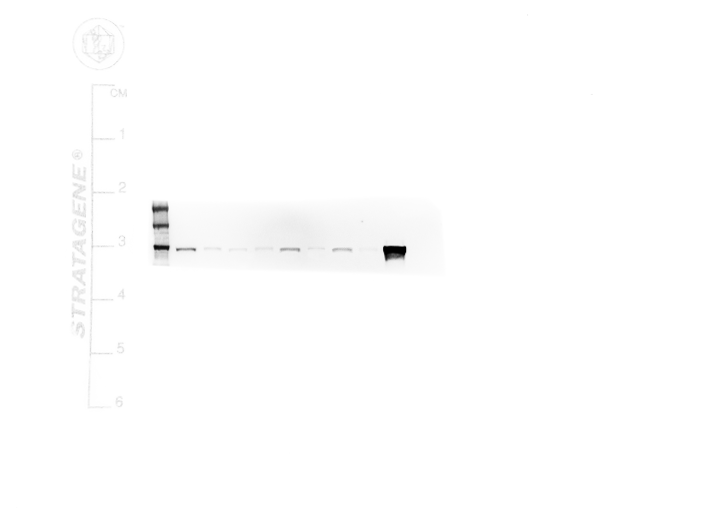

Supplement: Supplementary file 1 [file cancers-13-04993-s001.zip › cancers-1371721-supplementary for proof/cancers-1371721-original-images/Fig. 3/Fig 3 D ECAD.tif]

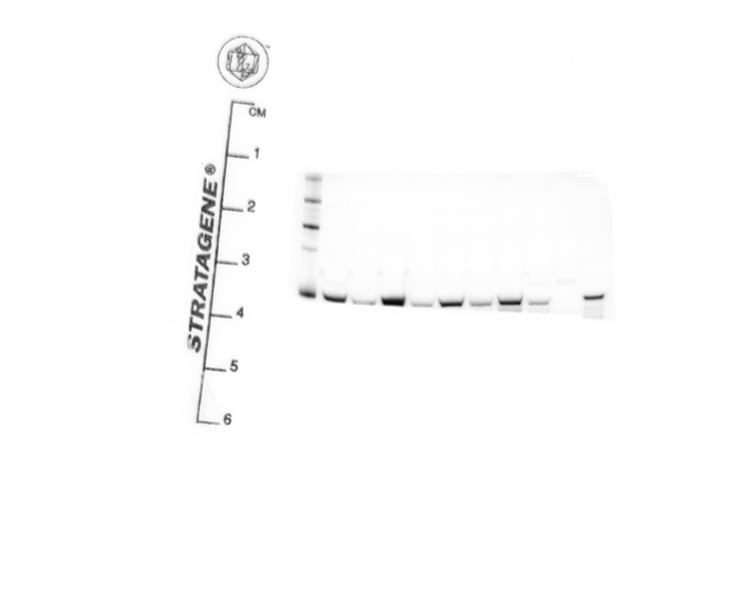

Supplement: Supplementary file 1 [file cancers-13-04993-s001.zip › cancers-1371721-supplementary for proof/cancers-1371721-original-images/Fig. 3/Fig 3 D Vim.tif]

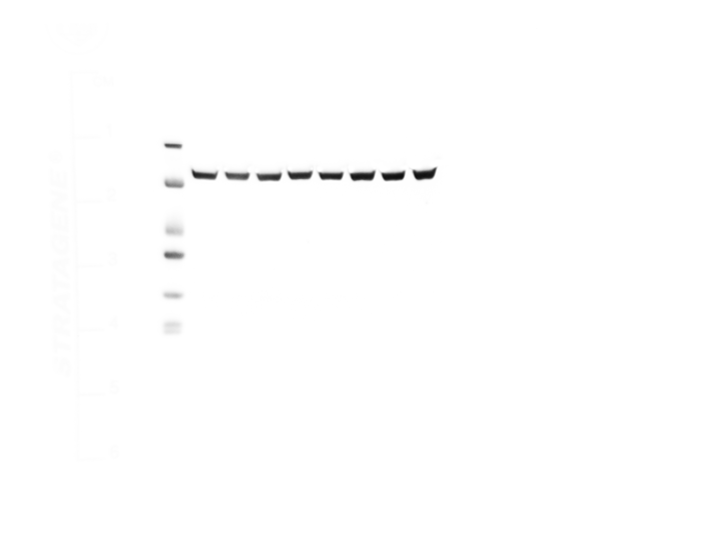

Supplement: Supplementary file 1 [file cancers-13-04993-s001.zip › cancers-1371721-supplementary for proof/cancers-1371721-original-images/Fig. 4/Fig. 4 A ACT.tif]

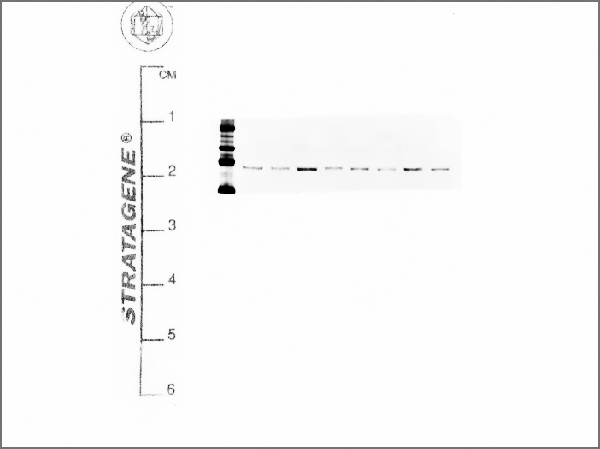

Supplement: Supplementary file 1 [file cancers-13-04993-s001.zip › cancers-1371721-supplementary for proof/cancers-1371721-original-images/Fig. 4/Fig. 4 A p-PKCEpsi.tif]

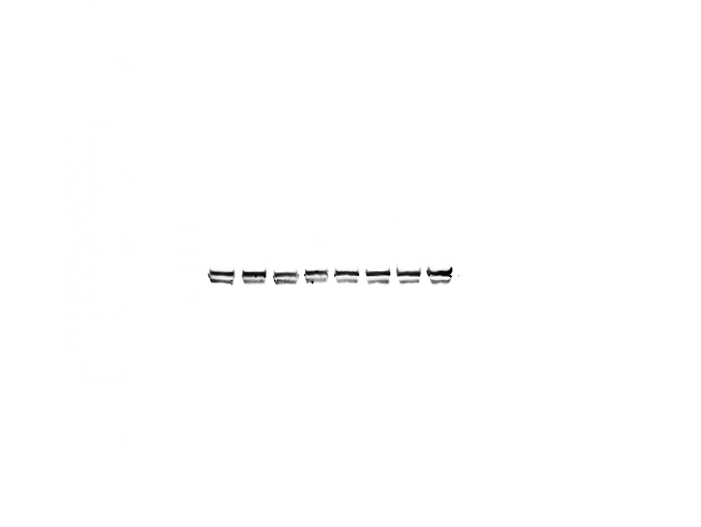

Supplement: Supplementary file 1 [file cancers-13-04993-s001.zip › cancers-1371721-supplementary for proof/cancers-1371721-original-images/Fig. 4/Fig. 4 A PKCEpsi Tot.tif]

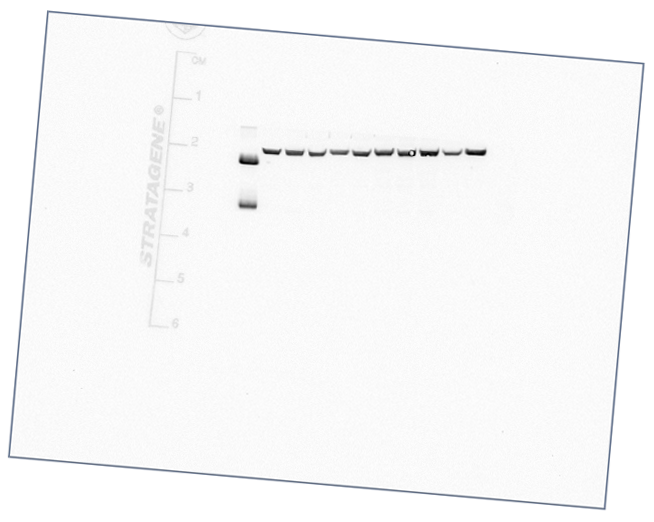

Supplement: Supplementary file 1 [file cancers-13-04993-s001.zip › cancers-1371721-supplementary for proof/cancers-1371721-original-images/Fig. 4/Fig. 4 C ACT.tif]

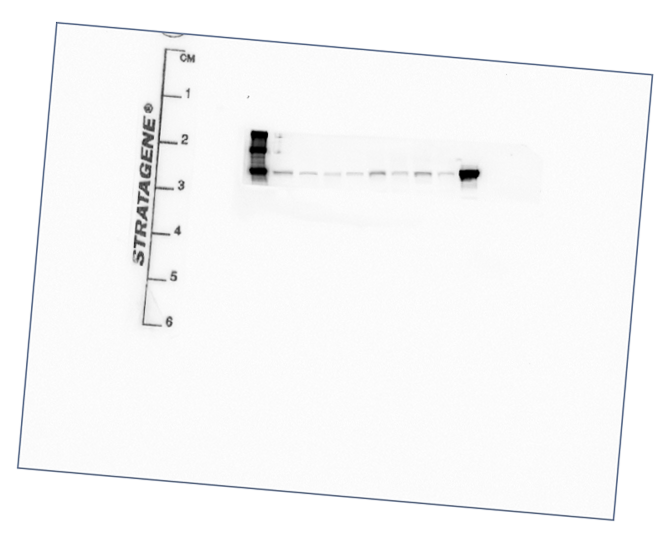

Supplement: Supplementary file 1 [file cancers-13-04993-s001.zip › cancers-1371721-supplementary for proof/cancers-1371721-original-images/Fig. 4/Fig. 4 C ECAD.tif]

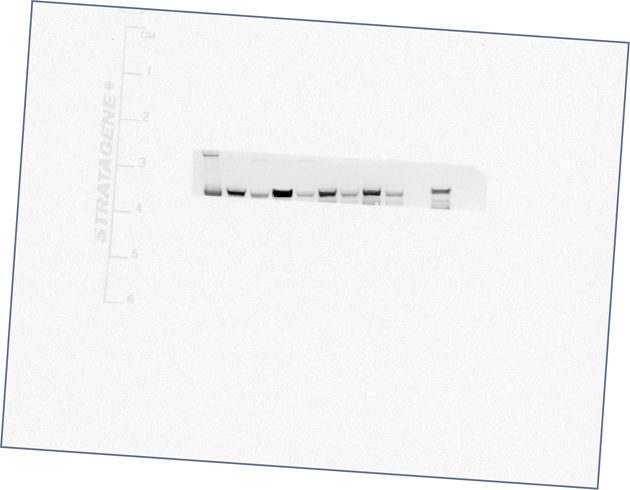

Supplement: Supplementary file 1 [file cancers-13-04993-s001.zip › cancers-1371721-supplementary for proof/cancers-1371721-original-images/Fig. 4/Fig. 4 C Vim.tif]

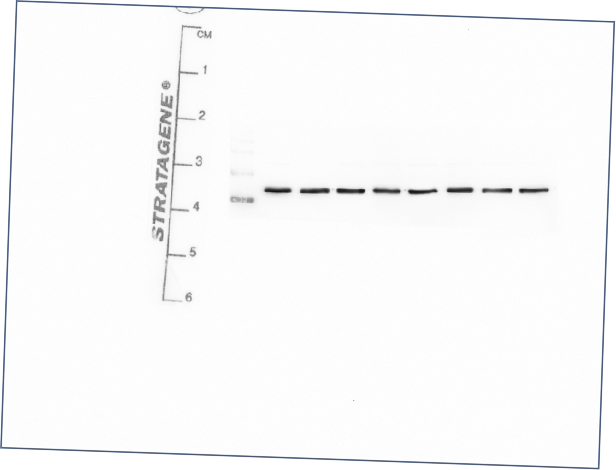

Supplement: Supplementary file 1 [file cancers-13-04993-s001.zip › cancers-1371721-supplementary for proof/cancers-1371721-original-images/Fig. 5/Fig. 5 A ACT (LC3).tif]

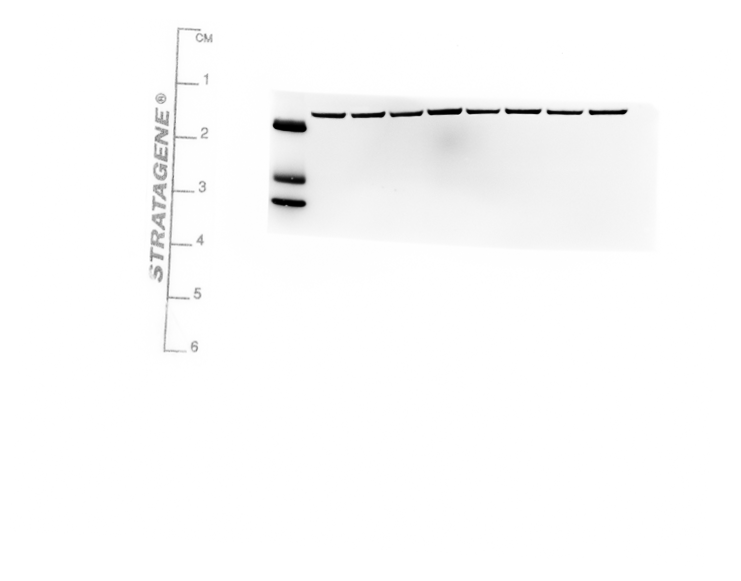

Supplement: Supplementary file 1 [file cancers-13-04993-s001.zip › cancers-1371721-supplementary for proof/cancers-1371721-original-images/Fig. 5/Fig. 5 A ACT (SQSTM1).tif]

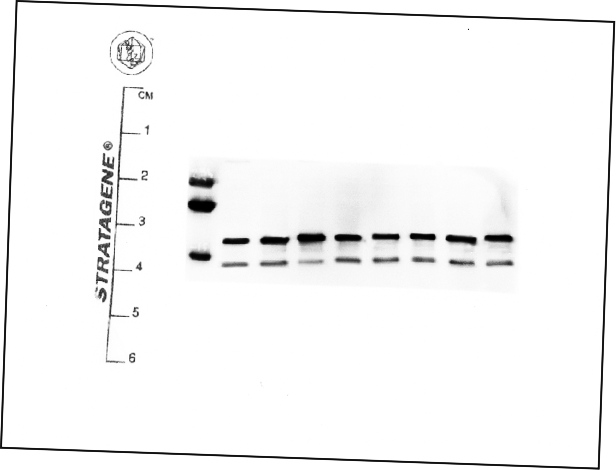

Supplement: Supplementary file 1 [file cancers-13-04993-s001.zip › cancers-1371721-supplementary for proof/cancers-1371721-original-images/Fig. 5/Fig. 5 A LC3.tif]

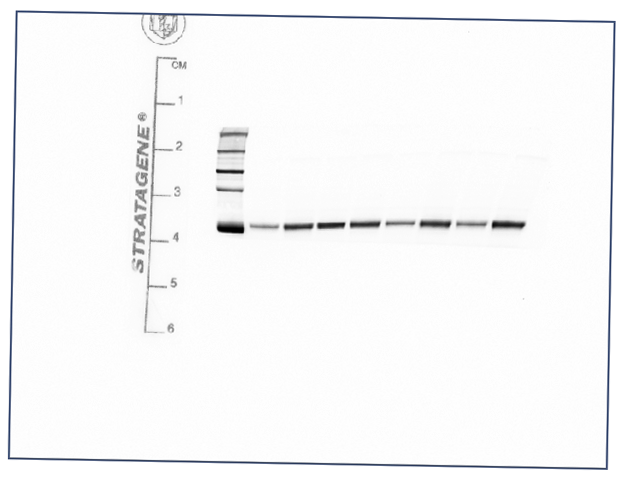

Supplement: Supplementary file 1 [file cancers-13-04993-s001.zip › cancers-1371721-supplementary for proof/cancers-1371721-original-images/Fig. 5/Fig. 5 A SQSTM1.tif]

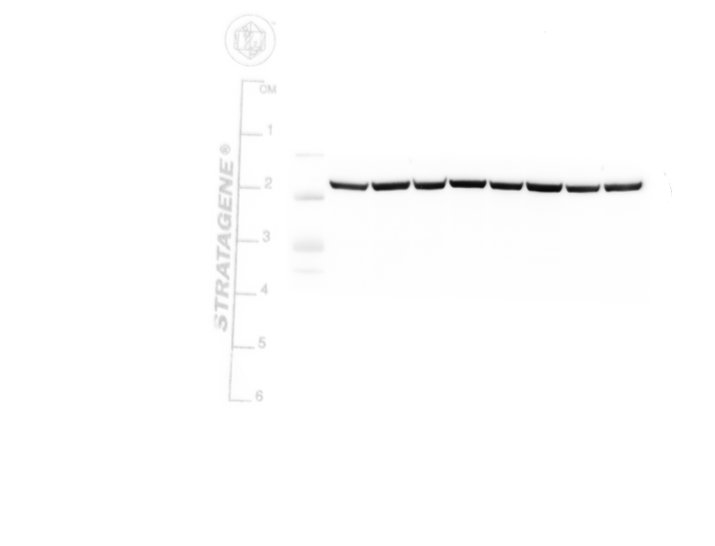

Supplement: Supplementary file 1 [file cancers-13-04993-s001.zip › cancers-1371721-supplementary for proof/cancers-1371721-original-images/Fig. 6/Fig. 6 A ACT.tif]

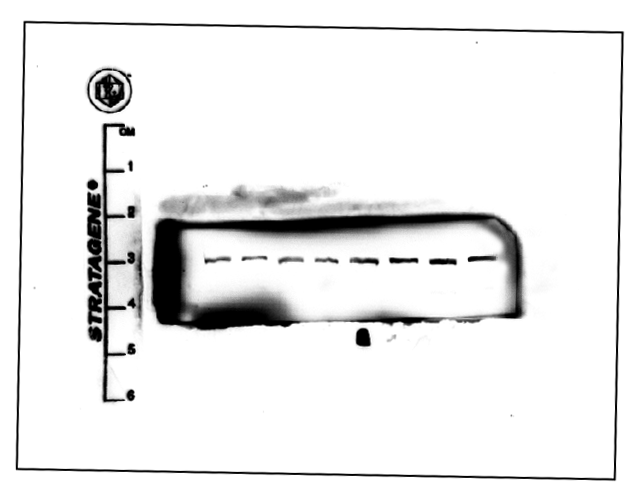

Supplement: Supplementary file 1 [file cancers-13-04993-s001.zip › cancers-1371721-supplementary for proof/cancers-1371721-original-images/Fig. 6/Fig. 6 A MTOR Tot.tif]

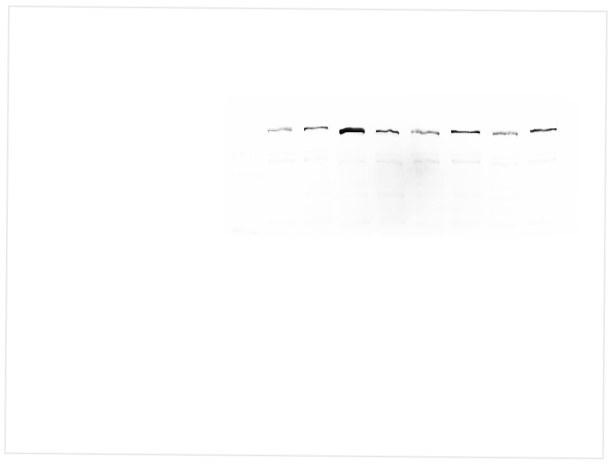

Supplement: Supplementary file 1 [file cancers-13-04993-s001.zip › cancers-1371721-supplementary for proof/cancers-1371721-original-images/Fig. 6/Fig. 6 A p-MTOR.tif]

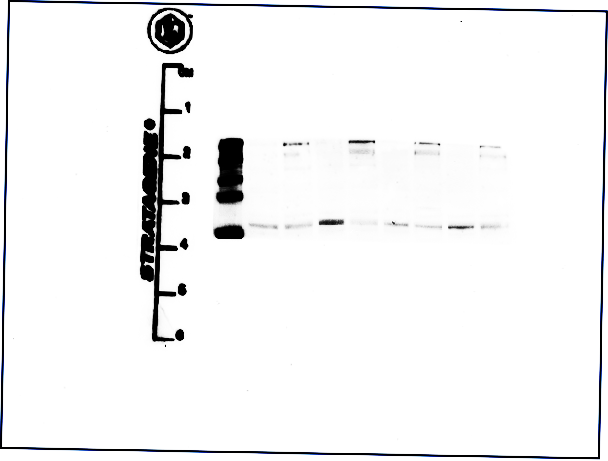

Supplement: Supplementary file 1 [file cancers-13-04993-s001.zip › cancers-1371721-supplementary for proof/cancers-1371721-original-images/Fig. 6/Fig. 6 A p-S6K.tif]

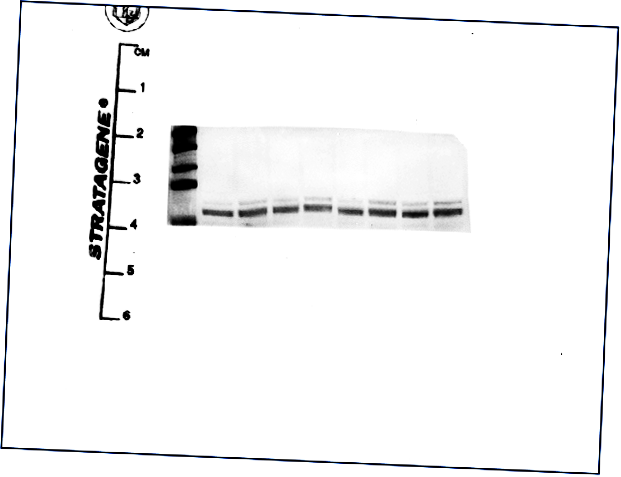

Supplement: Supplementary file 1 [file cancers-13-04993-s001.zip › cancers-1371721-supplementary for proof/cancers-1371721-original-images/Fig. 6/Fig. 6 A S6K Tot.tif]

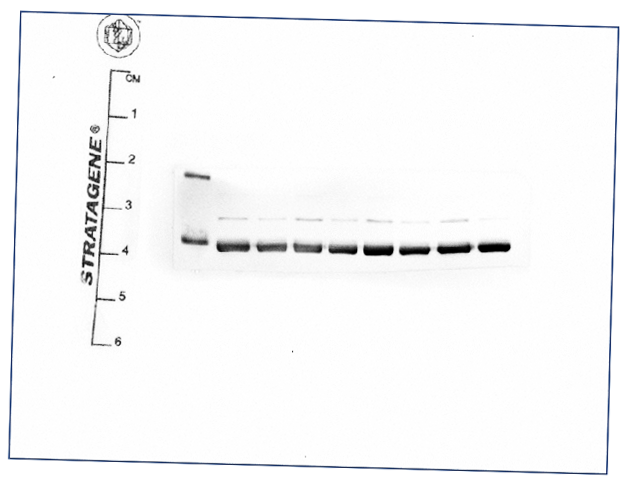

Supplement: Supplementary file 1 [file cancers-13-04993-s001.zip › cancers-1371721-supplementary for proof/cancers-1371721-original-images/Fig. 6/Fig. 6 A TUB.tif]

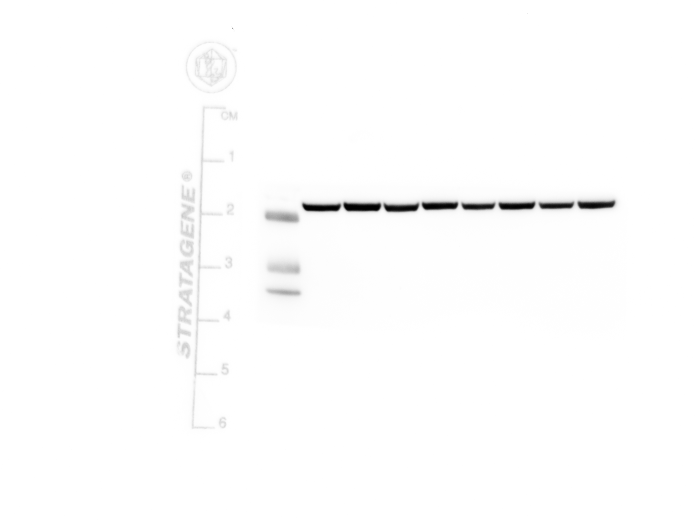

Supplement: Supplementary file 1 [file cancers-13-04993-s001.zip › cancers-1371721-supplementary for proof/cancers-1371721-original-images/Fig. 6/Fig. 6 B ACT.tif]

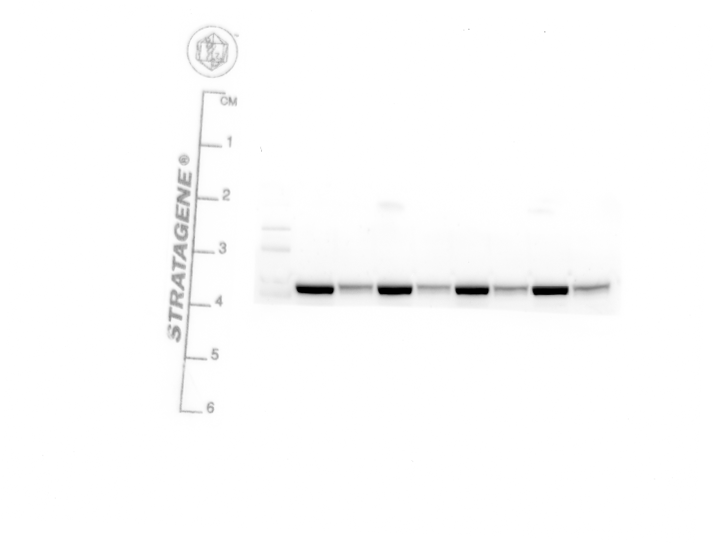

Supplement: Supplementary file 1 [file cancers-13-04993-s001.zip › cancers-1371721-supplementary for proof/cancers-1371721-original-images/Fig. 6/Fig. 6 B AKT Tot.tif]

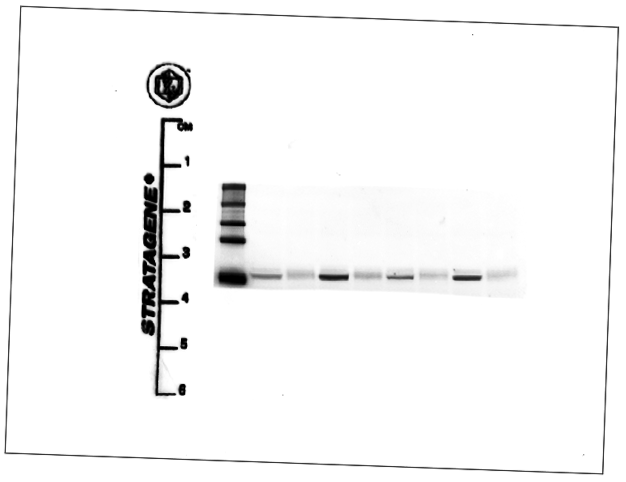

Supplement: Supplementary file 1 [file cancers-13-04993-s001.zip › cancers-1371721-supplementary for proof/cancers-1371721-original-images/Fig. 6/Fig. 6 B p-AKT.tif]

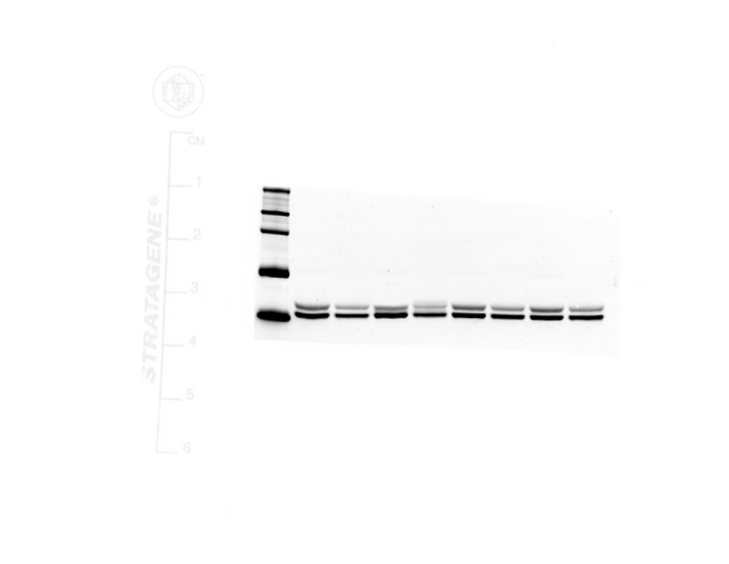

Supplement: Supplementary file 1 [file cancers-13-04993-s001.zip › cancers-1371721-supplementary for proof/cancers-1371721-original-images/Fig. 6/Fig. 6 C ERK Tot.tif]

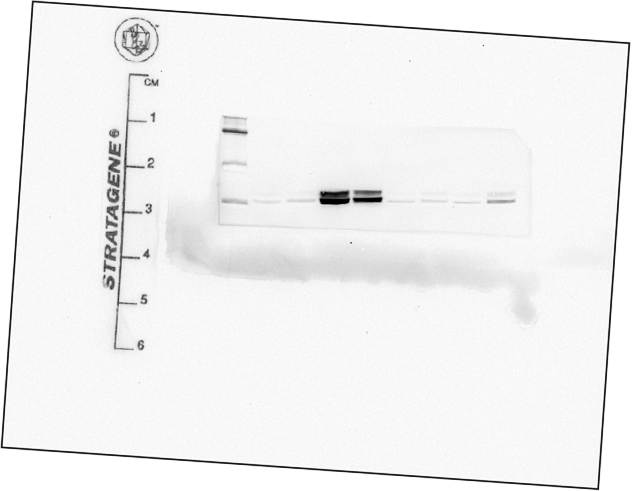

Supplement: Supplementary file 1 [file cancers-13-04993-s001.zip › cancers-1371721-supplementary for proof/cancers-1371721-original-images/Fig. 6/Fig. 6 C p-ERK.tif]

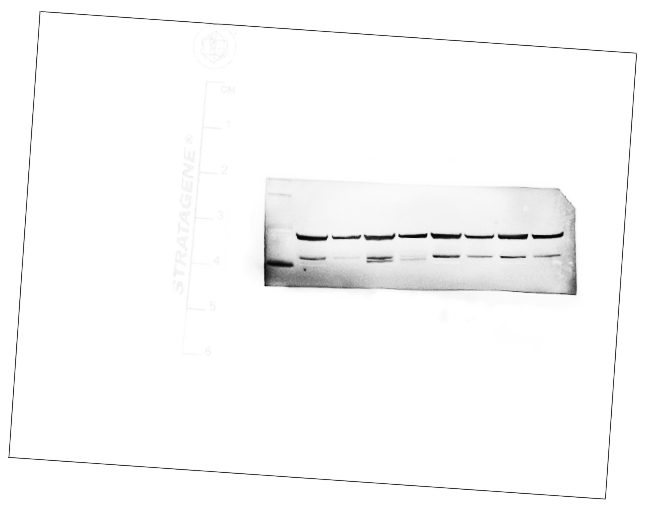

Supplement: Supplementary file 1 [file cancers-13-04993-s001.zip › cancers-1371721-supplementary for proof/cancers-1371721-original-images/Fig. 6/Fig. 6 C TUB.tif]

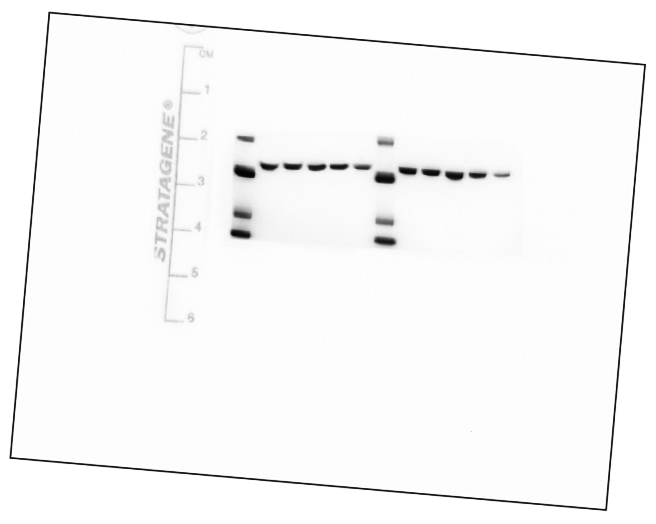

Supplement: Supplementary file 1 [file cancers-13-04993-s001.zip › cancers-1371721-supplementary for proof/cancers-1371721-original-images/Fig. S2/Fig. S2 ACT.tif]

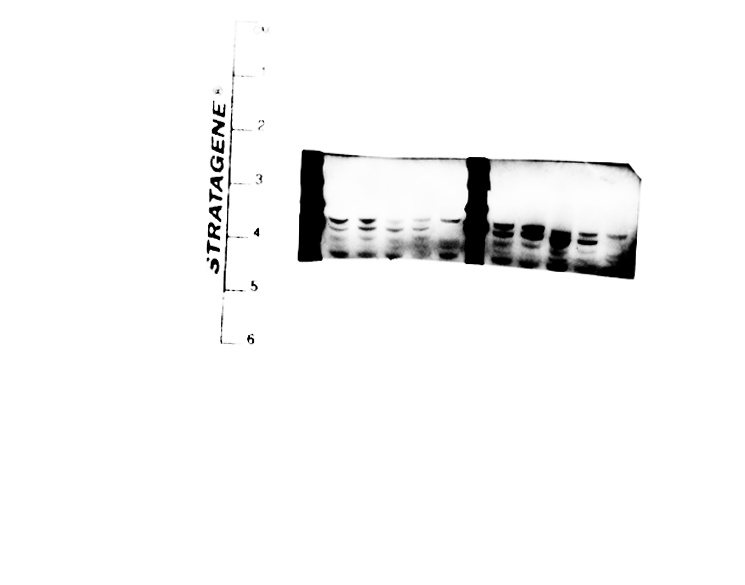

Supplement: Supplementary file 1 [file cancers-13-04993-s001.zip › cancers-1371721-supplementary for proof/cancers-1371721-original-images/Fig. S2/Fig. S2 PKCEpsi.tif]

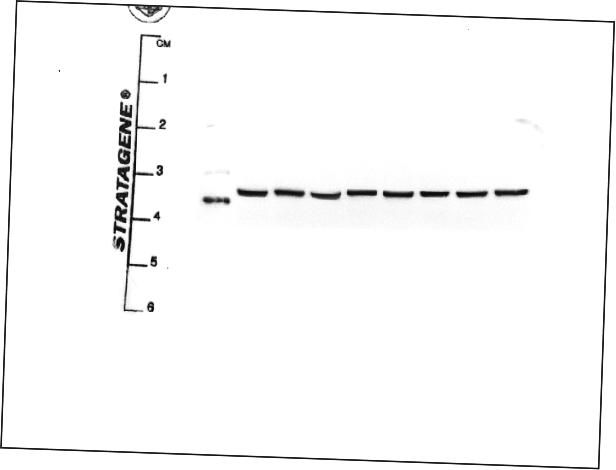

Supplement: Supplementary file 1 [file cancers-13-04993-s001.zip › cancers-1371721-supplementary for proof/cancers-1371721-original-images/Fig. S3/Fig S3 A ACT (LC3, shFGFR2).tif]

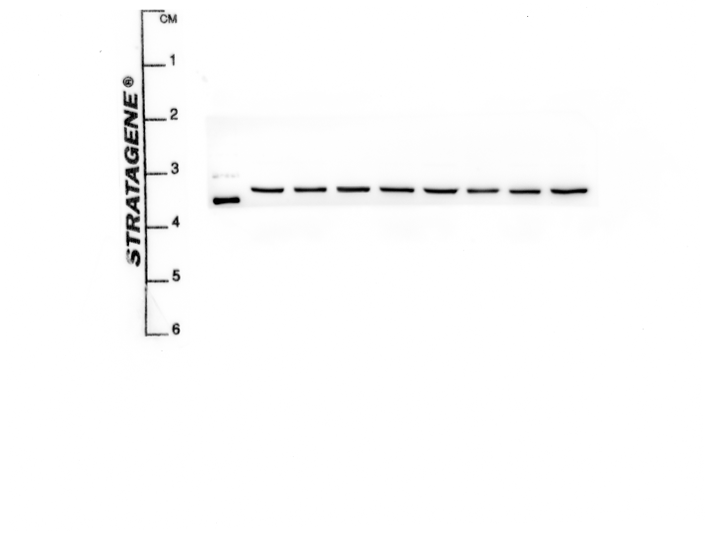

Supplement: Supplementary file 1 [file cancers-13-04993-s001.zip › cancers-1371721-supplementary for proof/cancers-1371721-original-images/Fig. S3/Fig S3 A ACT (LC3, SU5402).tif]

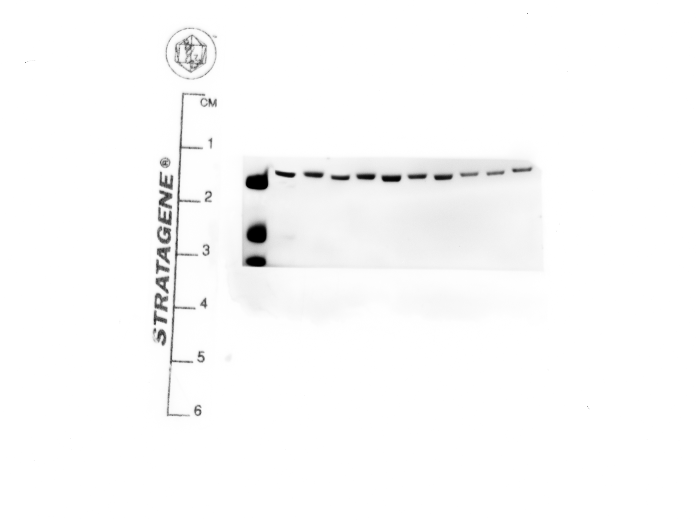

Supplement: Supplementary file 1 [file cancers-13-04993-s001.zip › cancers-1371721-supplementary for proof/cancers-1371721-original-images/Fig. S3/Fig S3 A ACT (SQSTM1, shFGFR2).tif]

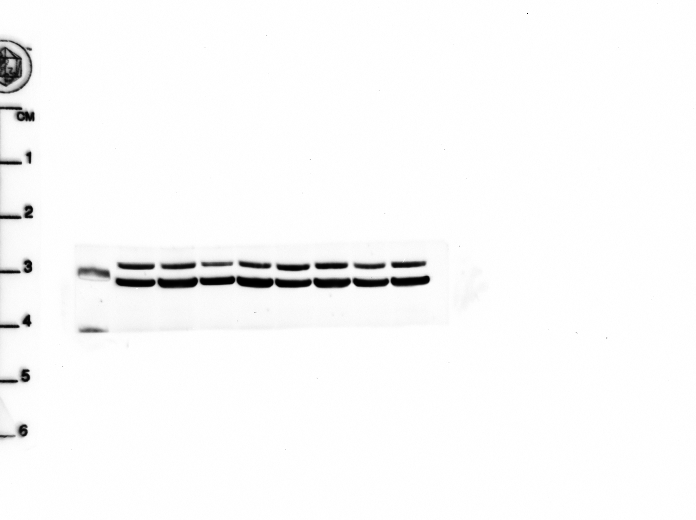

Supplement: Supplementary file 1 [file cancers-13-04993-s001.zip › cancers-1371721-supplementary for proof/cancers-1371721-original-images/Fig. S3/Fig S3 A ACT (SQSTM1, SU5402).tif]

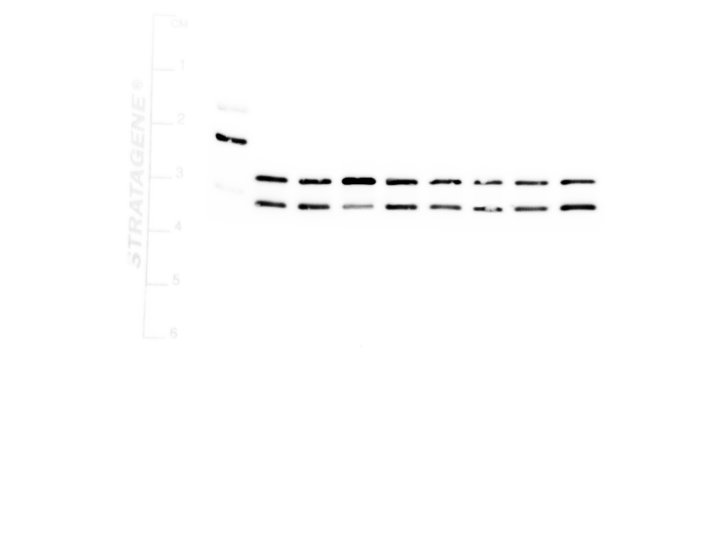

Supplement: Supplementary file 1 [file cancers-13-04993-s001.zip › cancers-1371721-supplementary for proof/cancers-1371721-original-images/Fig. S3/Fig S3 A LC3 (shFGFR2).tif]

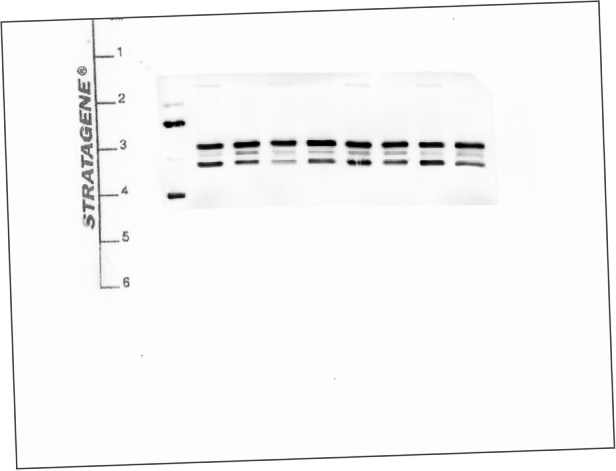

Supplement: Supplementary file 1 [file cancers-13-04993-s001.zip › cancers-1371721-supplementary for proof/cancers-1371721-original-images/Fig. S3/Fig S3 A LC3 (SU5402).tif]

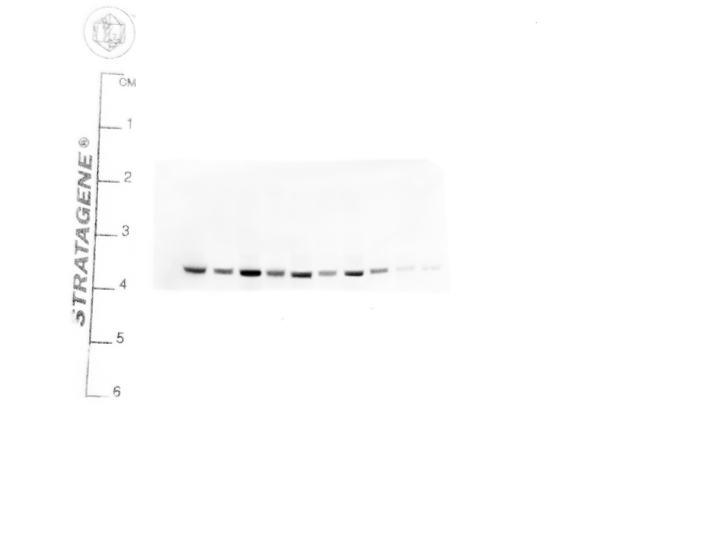

Supplement: Supplementary file 1 [file cancers-13-04993-s001.zip › cancers-1371721-supplementary for proof/cancers-1371721-original-images/Fig. S3/Fig S3 A SQSTM1 (shFGFR2).tif]

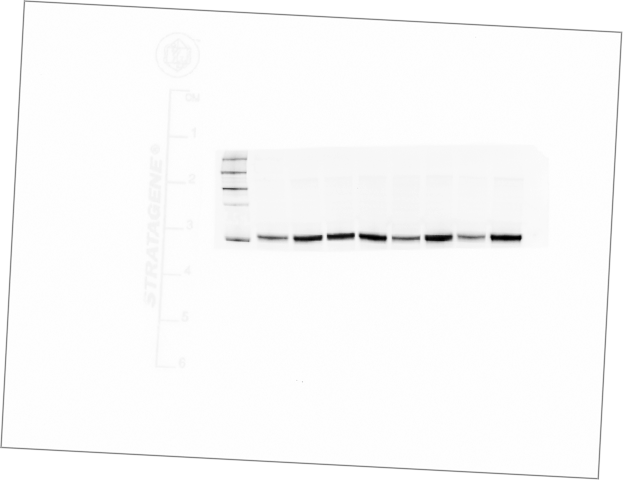

Supplement: Supplementary file 1 [file cancers-13-04993-s001.zip › cancers-1371721-supplementary for proof/cancers-1371721-original-images/Fig. S3/Fig S3 A SQSTM1 (SU5402).tif]
